# The evidence for improving housing to reduce malaria: a systematic review and meta-analysis

**DOI:** 10.1186/s12936-015-0724-1

**Published:** 2015-06-09

**Authors:** Lucy S Tusting, Matthew M Ippolito, Barbara A Willey, Immo Kleinschmidt, Grant Dorsey, Roly D Gosling, Steve W Lindsay

**Affiliations:** Department of Disease Control, London School of Hygiene & Tropical Medicine, London, WC1E 7HT UK; Department of Medicine, Division of Infectious Diseases, Johns Hopkins University School of Medicine, 1830 Building Room 450B, 600 North Wolfe Street, Baltimore, MD 21287 USA; Department of Infectious Disease Epidemiology, London School of Hygiene & Tropical Medicine, London, UK; Department of Medicine, University of California, San Francisco, CA 94143 USA; Global Health Group, University of California, San Francisco, CA 94105 USA; School of Biological and Biomedical Sciences, Durham University, Durham, DH1 3LE UK

**Keywords:** Malaria, *Plasmodium falciparum*, Vector, *Anopheles gambiae*, House, Eaves, Socio-economic

## Abstract

**Background:**

The global malaria burden has fallen since 2000, sometimes before large-scale vector control programmes were initiated. While long-lasting insecticide-treated nets and indoor residual spraying are highly effective interventions, this study tests the hypothesis that improved housing can reduce malaria by decreasing house entry by malaria mosquitoes.

**Methods:**

A systematic review and meta-analysis was conducted to assess whether modern housing is associated with a lower risk of malaria than traditional housing, across all age groups and malaria-endemic settings. Six electronic databases were searched to identify intervention and observational studies published from 1 January, 1900 to 13 December, 2013, measuring the association between house design and malaria. The primary outcome measures were parasite prevalence and incidence of clinical malaria. Crude and adjusted effects were combined in fixed- and random-effects meta-analyses, with sub-group analyses for: overall house type (traditional versus modern housing); screening; main wall, roof and floor materials; eave type; ceilings and elevation.

**Results:**

Of 15,526 studies screened, 90 were included in a qualitative synthesis and 53 reported epidemiological outcomes, included in a meta-analysis. Of these, 39 (74 %) showed trends towards a lower risk of epidemiological outcomes associated with improved house features. Of studies assessing the relationship between modern housing and malaria infection (*n* = 11) and clinical malaria (*n* = 5), all were observational, with very low to low quality evidence. Residents of modern houses had 47 % lower odds of malaria infection compared to traditional houses (adjusted odds ratio (OR) 0°53, 95 % confidence intervals (CI) 0°42–0°67, *p* < 0°001, five studies) and a 45–65 % lower odds of clinical malaria (case–control studies: adjusted OR 0°35, 95 % CI 0°20–0°62, *p* <0°001, one study; cohort studies: adjusted rate ratio 0°55, 95 % CI 0°36–0°84, *p* = 0°005, three studies). Evidence of a high risk of bias was found within studies.

**Conclusions:**

Despite low quality evidence, the direction and consistency of effects indicate that housing is an important risk factor for malaria. Future research should evaluate the protective effect of specific house features and incremental housing improvements associated with socio-economic development.

**Electronic supplementary material:**

The online version of this article (doi:10.1186/s12936-015-0724-1) contains supplementary material, which is available to authorized users.

## Background

Despite considerable advances in malaria control since 2000, with a 30 % fall in incidence in all age groups worldwide, the disease remains a major global public health problem with an estimated 198 million cases in 2013 [1]. Reductions have been achieved mainly through extensive long-lasting insecticide-treated net (LLIN) distribution and indoor residual spraying (IRS) campaigns. However, the future success of these interventions may be undermined by the spread of insecticide-resistant mosquitoes [2], creating a need for supplementary interventions not reliant on current insecticides. Interestingly, in some locations malaria has declined before intervention scale-up, suggesting additional causes of the reduction [3, 4]. Since malaria is a disease of poverty and the environment, there is increasing interest in the potential contribution of socio-economic development to malaria control [5], and in coordinating with sectors outside health, including agriculture, water and sanitation, education, city planning and housing, to meet long-term sustainable development goals [6].

Housing improvements, traditionally a key pillar of public health, remain underexploited in malaria control. Yet in sub-Saharan Africa (SSA), where up to 80-100 % of malaria transmission occurs indoors at night, the home can be a place of high risk [7]. House screening was the first intervention trialed in Italy after the link between malaria and mosquitoes was discovered [8]. Screening homes was subsequently shown to reduce malaria risk in India, South Africa and the USA [6] and better housing contributed to malaria elimination in the USA and Europe [9]. More recent studies indicate that well-built, modern housing can be protective in many tropical countries [10] and that simple features, including closed eaves (the gap between the top of the wall and the over-hanging roof), brick walls, tiled or metal roofs, or ceilings can reduce mosquito house entry [6]. In a randomized-controlled trial (RCT) in The Gambia, untreated door and window screens and closed eaves halved the prevalence of anaemia in children [11].

**Fig. 1:**
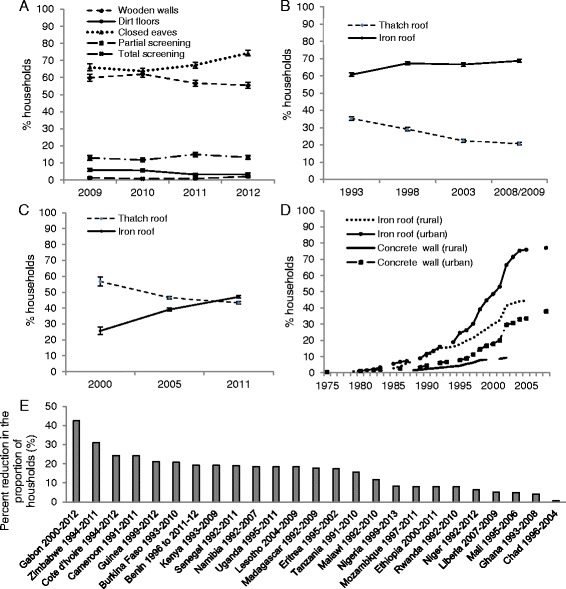
Changes in housing in sub-Saharan Africa, 1975–2012. Despite limited data, there is evidence that the quality of both urban and rural housing is improving in parts of SSA, including Bioko, Kenya, Ethiopia and Tanzania. **a**. Trends in housing in Bioko, Equatorial Guinea, 2009–2012 [18]. **b**. Proportion of homes with thatch and iron roofs in Kenya, 1993–2009 [33]. **c**. Proportion of homes with thatch and iron roofs in Ethiopia, 2000–2011 [33]. **d**. Estimated proportion of homes with concrete walls and iron roofs in Korogwe, Tanzania, 1975–2008 [34]. **e**. Percent reduction in the proportion of households with natural or rudimentary flooring in SSA (comparing earliest and latest available Demographic and Health Surveys (DHS); dates are shown for each country) [35]

Ninety per cent of malaria deaths in five year-olds occur in Africa, the economy of which is rapidly growing, with a 6 % annual increase in gross domestic product expected until 2025 [12]. Increased personal wealth is precipitating continent-wide housing improvements, such as the replacement of traditional thatch with metal and tiled roofs (Fig. 1). The expanding population, expected to triple to 1.23 billion by 2050, also needs accommodating, with an estimated 144 million new houses required by 2030 in rural areas alone [13]. This economic and cultural transition presents an opportunity to document and influence incremental housing improvements that might protect against malaria, and to build healthy homes.

Yet despite the historical precedent for improving housing to control malaria, few rigorously conducted studies exist. Furthermore, the evidence on housing and malaria has not been systematically characterized, with no specific evaluation of the size and consistency of the direction of effect, nor the quality of the evidence. The recent Multisectoral Action Framework for Malaria [14] emphasizes throughout the need for good housing, yet there is a paucity of evidence supporting this recommendation and uncertainty about how to select, scale-up and sustain intervention [6]. Here the potential for modern house construction to reduce malaria risk was evaluated. Specifically, the first systematic review and meta-analysis was conducted to assess whether ‘modern’ homes are associated with reduced exposure to infectious bites, malaria infection and clinical malaria in people of all ages in malaria-endemic regions, compared to ‘traditional’ homes. Since few intervention studies exist, observational study designs were also included. The study aimed first to characterize all published and unpublished data and second to assess the strength and quality of these data, in order to rigorously evaluate the evidence for the impact of housing improvements on malaria.

## Methods

Recommendations of the Preferred Reporting Items for Systematic Reviews and Meta-analysis of Observational Studies in Epidemiology groups were followed [15, 16]. The study is registered with the International Prospective Register of Systematic Reviews [17]. The study aimed to compare modern with traditional homes in any malaria-endemic settings. In SSA, traditional homes were considered to have mud walls, thatched roofs and earth floors, except in areas of exceptionally high rainfall including Equatorial Guinea, where concrete or wood is the basic wall material [18]. Traditional homes were considered to have mud or stone walls, thatched, wood or mud roofs, and earth floors in North Africa [19], wood or bamboo walls, thatched roofs and earth or wooden floors in Southeast and South Asia [20], and adobe or mud and wood walls, thatched roofs and earth floors in South America [21]. Universally, traditional homes were considered to have open eaves, no ceiling and no screening.

### Eligibility criteria

Studies were included with participants of any ages (excluding migrants, displaced people or military) and conducted in real (not experimental) houses, that compared modern with traditional house features and that measured any outcomes of interest. Both observational and intervention study designs were included: (1) case–control; (2) cohort; (3) cross-sectional studies; (4) randomized-controlled trials (RCTs); (5) controlled before-and-after studies, if arms were comparable at baseline and there was at least one unit per arm; (6) cross-over studies, if there were at least one unit per arm; and, (7) interrupted time-series studies. Studies were excluded if arm follow-up periods differed.

Epidemiological outcomes in human subjects were: clinical malaria (fever with parasitaemia confirmed by microscopy or rapid diagnostic test (RDT), in any age group); malaria infection (confirmed by microscopy or RDT, in any age group); and, anaemia in children aged under 11 years. Entomological outcomes were: entomological inoculation rate (EIR, the estimated number of bites by infectious mosquitoes per person per time period, measured directly using human baits or indirectly using light traps or other methods); human biting rate (HBR, the number of mosquitoes per person per time period); and, indoor density of adult vector mosquitoes (number of mosquitoes per house or person).

### Search strategy and data extraction

PubMed, Embase, LILACS, the Meta-Register of Controlled Trials, Cochrane Infectious Diseases Group Specialized Register, and Cochrane Central Register of Controlled Trials were searched with no language restrictions, using specified search terms (Additional file 1) to identify studies published from 1 January, 1900 to 13 December, 2013. The following databases were searched: US Armed Forces Pest Management Board online database (1900–1947) and proceedings of the MIM Pan-African Malaria Conferences (2005 and 2013), American Society of Tropical Medicine and Hygiene (2004–2013) and Society for Vector Ecology (2010–2012). Reference lists of identified studies were searched. Authors were contacted for additional references. LST and MI independently screened titles and abstracts before screening the full text of relevant studies using a standard form. Disagreements were resolved by SWL.

### Data extraction

Study characteristics (participants, sampling, exposures, comparisons, outcomes, study design, setting, sample size, follow-up period, vector(s), LLIN and IRS coverage, transmission intensity, and funding) were extracted by LST and a 10 % sub-sample randomly selected for validation (MI). Study authors were contacted for missing data.

### Risk of bias of and quality of evidence

Risk of bias for RCTs, controlled before-and-after studies, cross-over studies and interrupted time-series studies was assessed using the Effective Practice and Organization of Care (EPOC) tool [22], and for case–control, cohort and cross-sectional studies using the Newcastle-Ottawa Scale [23]. Risk of bias across studies (publication bias) was assessed using funnel plots and Egger’s test for funnel plot asymmetry [24]. Quality and strength of the evidence were evaluated for the main comparison (modern versus traditional homes) using the Grading of Recommendations, Assessment, Development and Evaluation (GRADE) approach [25].

### Data analysis

Analyses were structured first by house feature, second by outcome and third by study design. All eligible studies were included in a qualitative synthesis. Studies were also included in a quantitative analysis, comparing modern with traditional house features, if crude or adjusted odds ratios (ORs) or rate ratios (RRs) with 95 % confidence intervals (CI), or sufficient data to calculate crude effects, were reported. Specifically, epidemiological data were combined in meta-analysis and entomological data presented in tables. Analyses were done in Stata13 and RevMan5.

### Epidemiological data

Study effects were combined in the meta-analysis using the generic inverse variance method, which assigns each effect a weight equal to the inverse of its variance. Pooled ORs or RRs were calculated using fixed-effects meta-analysis where significant heterogeneity was not detected and random effects where significant heterogeneity was found (I^2^ > 50 %). Separate meta-analyses were done for crude and adjusted results.

### Entomological data

Data and study characteristics were presented in tables. Where no effect measure was reported the crude effect was calculated as the ratio of the outcomes in the treatment and control groups. Ninety-five per cent CIs were calculated by estimating the standard errors of the outcomes from their stated 95 % CI. Where 95 % CIs of outcomes were non-symmetrical, it was assumed that standard errors and CIs were calculated on log-transformed values.

## Results

### Search results

**Fig. 2:**
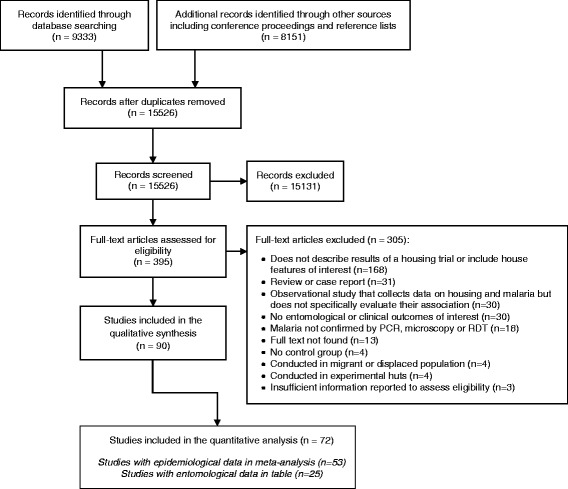
Study selection

The search yielded 15,526 studies after removing duplicates (Fig. 2). Ninety met the inclusion criteria, of which 18 were included in the qualitative synthesis only and 72 were included in the quantitative analysis (Additional file 2). Of these 72 studies, 53 reported epidemiological outcomes (included in the meta-analysis) and 25 reported entomological outcomes (presented in Tables).

### Study characteristics

The six intervention studies dated from 2009 to 2013. All were conducted in rural SSA, using house screening as the intervention. One study, a cluster RCT (cRCT) in The Gambia, collected both epidemiological and entomological outcomes [11] and was included in the meta-analysis. Five studies collected entomological data only: three pilot RCTs in Ethiopia, Mozambique and Tanzania [26–28], one randomized cross-over trial in The Gambia [29], and one non-randomized, cross-over trial in Tanzania [30]. The 84 observational studies, dating from 1935 to 2015, had cross-sectional (*n* = 39), cohort (*n* = 30), and case–control (*n* = 15) designs. These were conducted mainly in SSA (*n* = 58) and Asia (*n* = 13) and largely in rural settings (*n* = 62) (Additional file 2). In the 53 observational studies included in the meta-analysis, comparisons included modern versus traditional housing (*n* = 15); modern versus traditional wall (*n* = 22), roof (*n* = 18), and floor (*n* = 4) materials and closed versus open eaves (*n* = 11).

### Risk of bias and quality of the evidence

High risk of bias was found across numerous domains of the EPOC risk of bias for intervention studies, particularly for allocation concealment, length of follow-up and blinding (Additional file 3). Risk of bias within individual case–control, cross-sectional and cohort studies was generally high (Additional file 3). Across studies, there was no evidence of publication bias in the meta-analysis of house type and malaria infection (Additional file 4), with no evidence of funnel plot asymmetry (bias coefficient 0.52, 95 % CI −1.61 to 2.65, *p* = 0.60). There were insufficient studies to test for asymmetry in the meta-analysis of house type and clinical malaria. GRADE quality of the evidence for the main comparison, modern versus traditional housing, ranged from very low to low (Table 1).

**Table 1:** GRADE quality of evidence for the association between modern housing and clinical malaria outcomes

Outcomes	Summary of findings		Quality of the evidence					Overall quality of the evidence (GRADE)
	Relative effect (95 % CI)	No. participants (studies)	Risk of bias	Inconsistency	Indirectness	Imprecision	Publication bias	
Malaria infection: Case–control, cross-sectional and cohort studies (crude OR)	OR 0°46 (0°33–0°62)	22,700 (9 studies)	Serious^1^	No serious inconsistency^2^	No serious indirectness^3^	No serious imprecision^4^	Undetected^5^	LOW^1,2,3,4,5,6,7^ due to risk of bias, large effect
Malaria infection: Case–control, cross-sectional and cohort studies (adjusted OR)	OR 0°53 (0°42–0°67)	3949 (5 studies)	Serious^1^	No serious inconsistency^8^	No serious indirectness^9^	No serious imprecision^4^	Undetected^5^	VERY LOW^1,4,5,7,8,9,10^ due to risk of bias
Clinical malaria: Case–control and cross-sectional studies (crude OR)	OR 0°32 (0°19–0°54)	357 (1 study)	Serious^1^	No serious inconsistency^11^	Serious^12^	No serious imprecision^4^	Undetected^13^	VERY LOW^1,4,6,7,11,12,13^ due to risk of bias, indirectness, large effect
Clinical malaria: Case–control and cross-sectional studies (adjusted OR)	OR 0°35 (0°20–0°62)	357 (1 study)	Serious^1^	No serious inconsistency^11^	Serious^12^	No serious imprecision^4^	Undetected^13^	VERY LOW^1,4,6,7,11,12,13^ due to risk of bias, indirectness, large effect
Clinical malaria: Cohort studies (crude RR)	RR 0°22 (0°14–0°35)	1653 (3 studies)	Serious^1^	No serious inconsistency^14^	Serious^15^	No serious imprecision^4^	Undetected^13^	LOW^1,4,7,13,14,15,16^ due to risk of bias, indirectness, large effect
Clinical malaria: Cohort studies (adjusted RR)	RR 0°55 (0°36–0°84)	2237 (3 studies)	Serious^1^	No serious inconsistency^17^	Serious^15^	No serious imprecision^4^	Undetected^13^	VERY LOW^1,4,13,15,17,18^ due to risk of bias, indirectness

Patient or population: People of all ages living in malaria-endemic regions

Settings: East Timor, Egypt, Ethiopia, Greece, Malawi, Mexico, Sri Lanka, Tanzania, Thailand, Uganda and Yemen

Intervention: modern (versus traditional) housing

GRADE Working Group grades of evidence: High quality: Further research is very unlikely to change confidence in the estimate of effect. Moderate quality: Further research is likely to have an important impact on confidence in the estimate of effect and may change the estimate. Low quality: Further research is very likely to have an important impact on confidence in the estimate of effect and is likely to change the estimate. Very low quality: The estimate is very uncertain

^1^Downgraded by 1 for serious risk of bias: All studies were non-randomized and observational

^2^No serious inconsistency: All nine studies observed a protective effect of modern housing, compared to traditional housing. The smallest effect was a 28 % reduction in the odds of malaria infection

^3^No serious indirectness: These nine studies were conducted in a variety of sites, both urban and rural, in settings across sub-Saharan Africa, Asia and Europe. The findings are generalizable elsewhere

^4^No serious imprecision: The overall effect was statistically significant and clinically important

^5^Publication bias not detected: Egger's test for bias in crude results found no evidence funnel plot asymmetry (bias coefficient 0.52, 95 % CI −1.61 – 2.65, p = 0.60)

^6^Upgraded by 1 for large effect: OR lies within the range 0 to 0.5

^7^No evidence that residual confounding would reduce the demonstrated effect: no significant difference between crude and adjusted effects

^8^No serious inconsistency: All five studies observed a protective effect of modern housing, compared to traditional housing. The smallest effect was a 27 % reduction in the odds of malaria infection

^9^No serious indirectness: These five studies were conducted in a variety of sites, both urban and rural, in sub-Saharan Africa and Asia. The findings are generalizable elsewhere

^10^No large effect: Odds Ratio does not fall into the range 0 to 0.5

^11^No serious inconsistency: only one study

^12^Downgraded by 1 for indirectness: only one study was included, which was conducted in rural Mexico and the findings may not be generalizable elsewhere

^13^Publication bias not detected: insufficient studies to construct funnel plots

^14^No serious inconsistency: all three studies observed a protective effect of modern housing, compared to traditional housing. The smallest effect was a 53 % reduction in incidence of clinical malaria

^15^Downgraded by 1 for serious indirectness: all studies were conducted in rural sub-Saharan Africa. The results may not be generalizable to other settings

^16^Upgraded by 2 for very large effect: Rate ratio and 95 % confidence intervals lie within the range 0 to 0°5

^17^No serious inconsistency: all three studies observed a protective effect of modern housing, compared to traditional housing. The smallest effect was a 25 % reduction in the incidence of clinical malaria

^18^No large effect: RR does not fall into the range 0 to 0°5

### Modern versus traditional housing

**Fig. 3:**
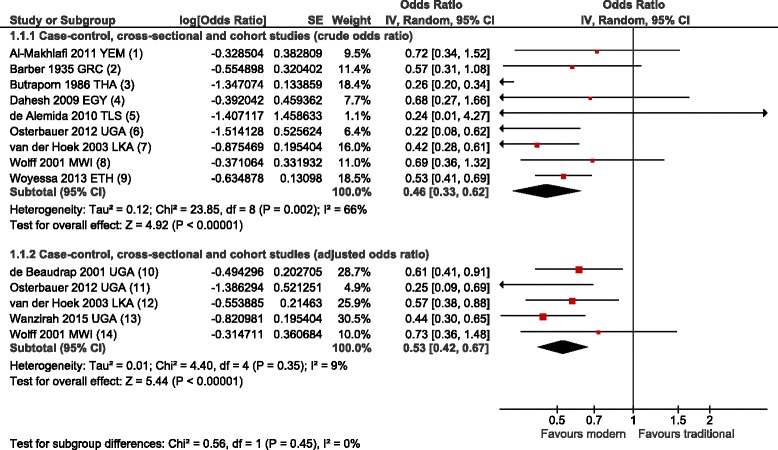
Meta-analysis of the association between modern housing and malaria infection. Pooled effects from random-effects meta-analyses for crude (1°1°1) and adjusted (1°1°2) results are shown. Studies are divided into sub-groups by study design. Error bars show 95 % CIs; df = degrees of freedom. 1. Al-Makhlafi 2011 YEM: Good *vs* poor house quality; 2. Barber 1935 GRC: Modern (tiled roof, ceiling) *vs* traditional (thatched roof, reed or no ceiling); 3. Butraporn 1935 THA: Permanent *vs* semi-permanent or temporary; 4. Dahesh 2009 EGY: Painted brick walls and cement ceilings *vs* mud walls and wood or mud ceilings; 5. de Alemida 2010 TLS: Complete *vs* incomplete house; 6. Osterbauer 2012 UGA: Modern (iron roof, burnt brick or cement walls and cement floor) *vs* traditional; 7. van der Hoek 2003 LKA: Modern (brick walls and permanent roof material) *vs* traditional (mud walls or thatched roof); 8. Wolff 2001 MWI: Modern *vs* traditional; 9. Woyessa 2013 ETH: Good *vs* dilapidated house, 10. de Beaudrap 2001 UGA: Brick walls and iron roof *vs* mud walls and thatched roof (OR adjusted for age, weight, socio-economic status, education, altitude, ITNs), 11. Osterbauer 2012 UGA: Modern (iron roof, burnt brick or cement walls and cement floor) *vs* traditional (OR adjusted for age, HIV-exposure, enrolment period, gender, mother's age, prophylaxis); 12. van der Hoek 2003 LKA: Modern (brick walls and permanent roof material) *vs* traditional (mud walls or thatched roof) (OR adjusted for age, gender, distance to stream, distance to cattle shed, coil use, ITNs, IRS); 13. Wanzirah 2015 UGA: Modern (cement, wood or metal wall; tiled or metal roof and closed eaves) *vs* traditional (OR adjusted for age, gender, study site, household wealth); 14. Wolff 2001 MWI: Modern *vs* traditional (OR adjusted for water source, occupation, education, malaria knowledge, waste disposal method)

**Fig. 4:**
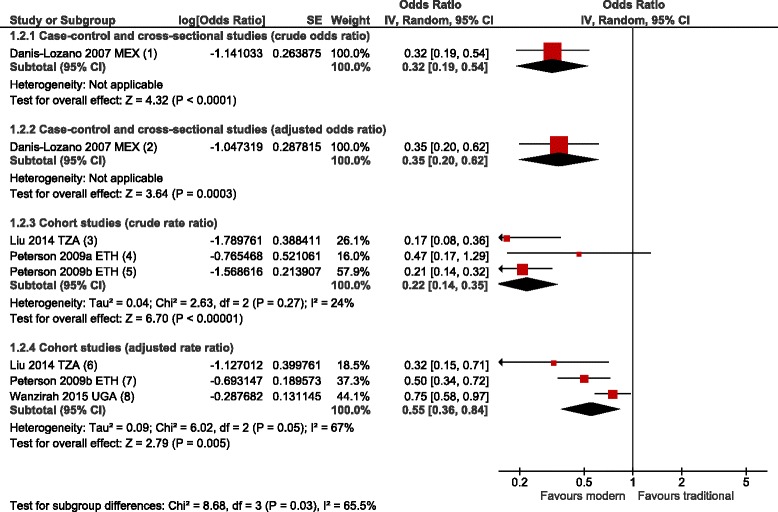
Meta-analysis of the association between modern housing and clinical malaria. Pooled effects from random-effects meta-analyses for crude (1°2°1; 1°2°3) and adjusted (1°2°2; 1°2°4) results are shown. Studies are divided into sub-groups by study design. Error bars show 95 % CIs; df = degrees of freedom. 1. Danis-Lozano 2007 MEX: House constructed with non-perishable *vs* perishable materials; 2. Danis-Lozano 2007 MEX: House constructed with non-perishable *vs* perishable materials (OR adjusted for occupation, village); 3. Liu 2014 TZA: Highest quintile of housing index compared to lowest quintile (based on roof, wall and floor material and presence of ceiling, eaves, screening); 4. Peterson 2009a ETH: Medium or good *vs* poor house construction; 5. Peterson 2009b ETH: Good *vs* poor house construction; 6. Liu 2014 TZA: Highest quintile of housing index compared to lowest quintile (based on roof, wall and floor material and presence of ceiling, eaves, screening) (RR adjusted for age, mother's education, wealth index, prophylaxis, socio-economic status, urban site, intermittent preventive treatment in infants (IPTi) trial arm); 7. Peterson 2009b ETH: Good *vs* poor house construction (RR adjusted for ITNs, vegetation, temperature, rainfall, larval densities); 8. Wanzirah 2015 UGA: Modern (cement, wood or metal wall; tiled or metal roof and closed eaves) *vs* traditional (RR adjusted for age, gender, study site, household wealth)

Residents of modern homes had lower odds of malaria infection than residents of traditional homes (crude OR 0°46, 95 % CI 0°33–0°62, *p* <0°001, nine studies, low quality evidence; adjusted OR 0°53, 95 % CI 0°42–0°67, *p* <0°001, five studies, very low quality evidence) (Fig. 3, Table 2). Modern homes were associated with lower odds and incidence rate of clinical malaria (case–control and cross-sectional studies: crude OR 0°32, 95 % CI 0°19–0°54, *p* <0°001, one study, very low quality evidence; adjusted OR 0°35, 95 % CI 0°20–0°62, *p* < 0°001, one study, very low quality evidence; cohort studies: crude RR 0°22, 95 % CI 0°14–0°35, *p* <0°001, three studies, low quality evidence; adjusted RR 0°55, 95 % CI 0°36–0°84, *p* = 0.005, three studies, very low quality evidence) (Fig. 4). In seven studies with entomological outcomes, modern housing was associated with no effect to a 66 % reduction in density of adult anophelines (Additional file 5).

**Table 2:** Summary of findings of meta-analyses of the association between specific house features and malaria

Comparison	Outcome	Study design	Total studies	Effect estimate
				(95 % CI)
1 Modern versus traditional housing	1.1 Malaria infection	1.1.1 Case–control, cross-sectional and cohort studies (crude OR)	9	0.46 [0.33, 0.62]
		1.1.2 Case–control, cross-sectional and cohort studies (adjusted OR)	5	0.53 [0.42, 0.67]
	1.2 Clinical malaria	1.2.1 Case–control and cross-sectional studies (crude OR)	1	0.32 [0.19, 0.54]
		1.2.2 Case–control and cross-sectional studies (adjusted OR)	1	0.35 [0.20, 0.62]
		1.2.3 Cohort studies (crude RR)	3	0.22 [0.14, 0.35]
		1.2.4 Cohort studies (adjusted RR)	3	0.55 [0.36, 0.84]
2 Screening^1^	2.1 Anaemia in children aged 0–11 years	2.1.1 Randomized controlled trials (adjusted OR)	1	0.52 [0.34, 0.80]
		2.1.2 Case–control, cross-sectional and cohort studies (crude OR)	2	0.65 [0.33, 1.30]
		2.1.3 Case–control, cross-sectional and cohort studies (adjusted OR)	1	0.56 [0.24, 1.27]
	2.2 Malaria infection	2.2.1 Randomized controlled trials (adjusted OR)	1	0.95 [0.63, 1.43]
		2.2.2 Case–control, cross-sectional and cohort studies (crude OR)	5	0.35 [0.13, 0.98]
		2.2.3 Case–control, cross-sectional and cohort studies (adjusted OR)	2	0.93 [0.82, 1.05]
	2.3 Clinical malaria	2.3.1 Case–control and cross-sectional studies (crude OR)	1	1.16 [0.82, 1.64]
		2.3.2 Cohort studies (crude RR)	5	0.71 [0.49, 1.04]
		2.3.3 Cohort studies (adjusted RR)	3	0.56 [0.46, 0.67]
3 Main wall material^2^	3.1 Anaemia in children aged 0–11 years	3.1.1 Case–control, cross-sectional and cohort studies (crude OR)	1	0.58 [0.33, 1.02]
		3.1.2 Case–control, cross-sectional and cohort studies (adjusted OR)	1	0.57 [0.29, 1.12]
	3.2 Malaria infection	3.2.1 Case–control, cross-sectional and cohort studies (crude OR)	12	0.57 [0.42, 0.78]
		3.2.2 Case–control, cross-sectional and cohort studies (adjusted OR)	7	0.73 [0.62, 0.85]
	3.3 Clinical malaria	3.3.1 Case–control and cross-sectional studies (crude OR)	7	0.63 [0.43, 0.93]
		3.3.2 Case–control and cross-sectional studies (adjusted OR)	1	0.16 [0.06, 0.44]
		3.3.3 Cohort studies (crude RR)	1	2.07 [1.18, 3.63]
		3.3.4 Cohort studies (adjusted RR)	2	1.05 [0.48, 2.30]
4 Main roof material^2^	4.1 Anaemia in children aged 0–11 years	4.1.1 Case–control, cross-sectional and cohort studies (crude OR)	1	0.71 [0.45, 1.12]
	4.2 Malaria infection	4.2.1 Case–control, cross-sectional and cohort studies (crude OR)	9	0.64 [0.48, 0.86]
		4.2.2 Case–control, cross-sectional and cohort studies (adjusted OR)	6	0.83 [0.64, 1.08]
	4.3 Clinical malaria	4.3.1 Case–control and cross-sectional studies (crude OR)	4	0.86 [0.48, 1.53]
		4.3.2 Case–control and cross-sectional studies (adjusted OR)	1	0.30 [0.13, 0.66]
		4.3.3 Cohort studies (crude RR)	2	0.59 [0.52, 0.67]
		4.3.4 Cohort studies (adjusted RR)	3	0.79 [0.70, 0.88]
5 Main floor material^2^	5.1 Anaemia in children aged 0–11 years	5.1.1 Case–control, cross-sectional and cohort studies (crude OR)	1	0.78 [0.45, 1.34]
	5.2 Malaria infection	5.2.1 Case–control, cross-sectional and cohort studies (crude OR)	1	1.20 [0.69, 2.09]
		5.2.2 Case–control, cross-sectional and cohort studies (adjusted OR)	2	0.74 [0.57, 0.96]
	5.3 Clinical malaria	5.3.1 Case–control and cross-sectional studies (crude OR)	1	0.19 [0.06, 0.57]
		5.3.2 Cohort studies (adjusted RR)	1	0.81 [0.62, 1.06]
6 Eaves^3^	6.1 Malaria infection	6.1.1 Case–control, cross-sectional and cohort studies (crude OR)	4	0.70 [0.58, 0.84]
		6.1.2 Case–control, cross-sectional and cohort studies (adjusted OR)	3	0.78 [0.70, 0.87]
	6.2 Clinical malaria	6.2.1 Case–control and cross-sectional studies (crude OR)	5	0.76 [0.55, 1.07]
		6.2.2 Case–control and cross-sectional studies (adjusted OR)	1	0.53 [0.36, 0.80]
		6.2.3 Cohort studies (crude RR)	1	0.75 [0.50, 1.12]
		6.2.4 Cohort studies (adjusted RR)	2	0.71 [0.46, 1.11]
7 Ceiling^4^	7.1 Clinical malaria	7.1.1 Case–control and cross-sectional studies (crude OR)	3	0.68 [0.56, 0.83]
		7.1.2 Case–control and cross-sectional studies (adjusted OR)	1	0.65 [0.46, 0.93]
8 Elevation^5^	8.1 Malaria infection	8.1.1 Case–control and cross-sectional studies (crude OR)	1	1.00 [1.00, 1.00]

^1^Screened versus unscreened; ^2^ Modern versus traditional main wall, roof and floor material: traditional homes were considered to have mud walls, a thatched roof and earth floors in sub-Saharan Africa (except in areas of high rainfall including Equatorial Guinea, where the basic wall material is typically concrete or wood [18]); mud or stone walls, a thatched, wood or mud roof and earth floors in North Africa; wood or bamboo walls, a thatched roof and wooden (stilted) floors in Southeast Asia; mud or wood walls, a thatched roof and earth or wooden (stilted) floors in South Asia; adobe or mud and wood walls, a thatched roof and earth floors in South America. ^3^ Closed versus open eaves; ^4^ Presence versus absence of a ceiling; ^5^ Elevated versus non-elevated houses

### House screening

In one cRCT in The Gambia, full or ceiling screening reduced anaemia in children by 48 % (adjusted OR 0°52, 95 % CI 0°34–0°80, *p* = 0°003), with no effect on malaria infection. Screening was not consistently associated with lower odds of anaemia in children or malaria infection in seven case–control, cross-sectional and cohort studies, but was associated with a lower incidence of clinical malaria in three cohort studies (Table 2).

### Modern versus traditional wall, roof and floor materials

Modern wall materials were associated with an approximately quarter reduction in the odds of malaria infection, although results were inconsistent for incidence of clinical malaria. Modern roof materials were not consistently associated with reduced odds of infection, but were associated with up to a two thirds reduction in the incidence of clinical malaria. There was inconsistent evidence that modern floor materials gave protection against any epidemiological outcome (Table 2).

### Eaves, ceilings and house elevation

Closed eaves were associated with a quarter reduction in the odds of malaria infection and a quarter to a half reduction in clinical malaria in five case–control and cross-sectional studies and two cohort studies. The presence (versus absence) of a ceiling was associated with a third reduction in the odds of clinical malaria. In one cross-sectional study, house elevation was not associated with the odds of malaria infection (Table 2).

## Discussion

In this systematic review and meta-analysis to assess whether modern housing is associated with a lower risk of malaria than traditional housing, 84 observational and six intervention studies were included. In eleven case–control, cohort and cross-sectional studies in East Timor, Egypt, Ethopia, Greece, Malawi, Sri Lanka, Thailand, Uganda, and Yemen, the odds of malaria infection were halved in modern versus traditional homes. In one case–control study in Mexico, the odds of clinical malaria were reduced by two thirds. In four cohort studies in Ethiopia, Tanzania and Uganda, the incidence of clinical malaria was halved in modern versus traditional homes.

Although house screening was the first intervention trialled against malaria [8], few intervention studies have rigorously evaluated the effect of housing on malaria. Observational studies were therefore also included here, which were most likely subject to selection and measurement bias, low comparability between groups, residual confounding by wealth [5], and geographical clustering of socio-economic status, house design and malaria. Although we found no evidence of publication bias across studies, we had limited power to detect publication bias due to the relatively small number of studies included [24]. Therefore it is highly possible that publication bias, selective outcome reporting, small-study effects, or selective analysis reporting were present across studies. Overall GRADE quality of evidence was judged to be ‘very low’ to ‘low’, indicating considerable uncertainty in the estimated effects. Despite this, the relative consistency of the size and direction of effect across studies and settings indicates some protection by modern housing, compared to traditional homes, in urban and rural settings in Africa, Asia and South America. Specifically, wall and roof materials other than traditional wood, mud and thatch, and modern house designs encompassing closed eaves, screened doors and windows, and ceilings, may help reduce mosquito house entry and malaria transmission, and therefore merit further field evaluation.

Good housing can help protect by blocking the entry routes of malaria vectors, which vary by species and region. Overall, the reduced prevalence and incidence of malaria in modern versus traditional homes indicates that this classification was a good proxy for overall ease of entry by mosquitoes across different settings. Closed eaves are likely to be protective in sub-Saharan Africa since the primary African vector *Anopheles gambiae s.l.* locates hosts by following odour plumes close to the ground and flying upwards when a vertical surface is reached. Open eaves then funnel mosquitoes inside [29]. The presence of a ceiling possibly replicates the protective effect of closed eaves. Conversely, eaves may be less important in South East Asia, where vector entry differs. For example, open verandas are a key feature for house entry by *Anopheles philippinensis* in Laos PDR [20]. Screening doors and windows can help to directly block vector entry, while modern wall and roof materials may contain fewer gaps, alter the attractiveness of the interior environment to mosquitoes or provide fewer resting sites for mosquitoes than traditional materials such as mud or thatch. It has also been hypothesized that metal-roofed homes are hotter and less conductive for mosquito survival; in Tanzania, the mean physiological age of vectors and sporozoite rate was observed to be lower in more modern versus traditional villages [31]. Understanding the mechanism of protection of different house features against individual vectors is important for identifying potential synergy or discordance with IRS and LLINs.

Housing is incrementally improving across much of SSA as living standards increase. The present analysis suggests that modern house improvements should be further evaluated in relation to malaria, in addition to specific house modifications including screening. If effective, housing could help reduce reliance on insecticides by providing an additional and more permanent intervention where LLINs and IRS are compromised by behavioural and physiological resistant vectors [2]. Furthermore, since malaria has declined in many African countries, often prior to specific intervention, further research to evaluate the contribution of housing improvements and the expansion of urban environments less conducive to malaria transmission is advocated [5]. Improving the home environment aligns with integrated vector management (IVM) and may help protect against other vector-borne diseases, such as filariasis, cutaneous leishmaniasis, Japanese encephalitis, and dengue, where vectors enter houses [32], and diarrhoeal disease, through better water, sanitation and hygiene (WASH). Since global housing programmes are key strategies of UN-HABITAT and Habitat for Humanity among other organisations, a pipeline for building malaria-safe homes already exists.

Improving housing will not be equally effective everywhere, since outdoor transmission can limit the efficacy of interventions centered on the home. Future research should address questions of equity by investigating whether mosquitoes diverted from improved houses may increase exposure among unprotected neighbours. Potentially damaging health effects must also be considered, such as an increased risk of respiratory diseases if airflow is restricted in the presence of certain cooking fuels. It is also shown here that the evidence base for housing needs strengthening, with only one intervention study that measured clinical outcomes [11]. Therefore further small-scale experimental studies to pinpoint exactly which house features can reduce vector entry cost effectively in different settings, RCTs with epidemiological outcomes, and concurrent studies addressing how to incorporate protective features into local house designs and building regulations are needed.

## Conclusions

Despite low quality evidence, the direction and consistency of effects indicate that housing is an important risk factor for malaria. Future research should evaluate the protective effect of both specific house features and incremental housing improvements associated with socio-economic development. Investment in such research and in housing programmes should be considered a natural component of malaria control efforts and a close complement to IVM and WASH as part of long-term, sustainable development.

## Additional files

Additional file 1:
**Search strategy.**
Additional file 2:
**Characteristics of included studies.**
Additional file 3:
**Risk of bias assessment for studies included in the quantitative analysis.**
Additional file 4:
**Funnel plots to assess publication bias.**
Additional file 5:
**Association between house construction and entomological outcomes.**

## Abbreviations

CI: Confidence interval
EIR: Entomological inoculation rate
EPOC: Cochrane effective practice and organization of care
GRADE: Grading of recommendations, assessment, development and evaluation
HBR: Human biting rate
IRS: Indoor residual spraying
IVM: Integrated vector management
LLIN: Long-lasting insecticidal net
OR: Odds ratio
RCT: Randomized controlled trial
RDT: Rapid diagnostic test
RR: Rate ratio
SSA: Sub-Saharan Africa
WASH: Water, sanitation and hygiene
