# Supplementary material for: The evidence for improving housing to reduce malaria: a systematic review and meta-analysis
Source: Malar J. 2015 Jun 9;14:209. doi: 10.1186/s12936-015-0724-1 (PMC4460721; doi:10.1186/s12936-015-0724-1)
Supplement: Additional file 1: — Search strategy. [file 12936_2015_724_MOESM1_ESM.pdf]

Tusting LS, Ippolito M, et al. The evidence for improving housing to reduce malaria: a systematic review and meta-analysis.

Additional File 1. Search strategy in PubMed (search date December 13, 2013)

1. Malaria (Medical Subject Headings (MeSH) term)
2. Anopheles (MeSH term)
3. Mosquito Control (MeSH term)
4. Plasmodium (MeSH term)
5. Disease vectors (MeSH term)
6. Insect vectors (MeSH term)
7. Entomology (MeSH term)
8. Malaria (text word)
9. Mosquito* (text word)
10. Anophel* (text word)
11. Entomologic* (text word)
12. Parasitemi* (text word)
13. Parasitaemi* (text word)
14. Plasmodium (text word)
15. 1 or 2 or 3 or 4 or 5 or 6 or 7
16. 8 or 9 or 10 or 11 or 12 or 13 or 14
17. 15 or 16
18. Housing (MeSH term)
19. Architecture as topic (MeSH term)
20. Hous* (text word)
21. Home (text word)
22. Homes (text word)
23. Hut (text word)
24. Huts (text word)
25. Shelter (text word)
26. Shelters (text word)
27. Building* (text word)
28. Dwelling* (text word)
29. Eave* (text word)
30. Wall (text word)
31. Walls (text word)
32. Air brick* (text word)
33. Airbrick* (text word)
34. Roof (text word)
35. Roofing (text word)
36. Door (text word)
37. Doors (text word)
38. Window* (text word)
39. Ceiling* (text word)
40. Stilt (text word)
41. Stilts (text word)
42. 18 or 19
43. 20 or 21 or 22 or 23 or 24 or 25 or 26 or 27 or 28
44. 29 or 30 or 31 or 32 or 33 or 34 or 35 or 36 or 37 or 38 or 39 or 40 or 41
45. 42 or 43 or 44
46. 17 and 45
