# Supplementary material for: The evidence for improving housing to reduce malaria: a systematic review and meta-analysis
Source: Malar J. 2015 Jun 9;14:209. doi: 10.1186/s12936-015-0724-1 (PMC4460721; doi:10.1186/s12936-015-0724-1)
Supplement: Additional file 2: — Characteristics of included studies. [file 12936_2015_724_MOESM2_ESM.pdf]

Tusting LS, Ippolito M, et al. The evidence for improving housing to reduce malaria: a systematic review and meta-analysis.

Additional File 2. Characteristics of studies

a. Characteristics of intervention studies included in the quantitative analysis (n=6)

Reference	Kampango 2013[1]	Kirby 2009[2]	Massebo 2013[3]	Mng'ong'o 2011[4]	Njie 2009[5]	Ogoma 2010[6]
**Trial design**	Randomised controlled study (pilot)	Randomised controlled study	Randomised controlled study (pilot)	Randomised controlled study (pilot)	Randomised cross–over study (pilot)	Non–randomized cross–over study (pilot)
**Age**	n/a	6 months to 10 years	n/a	n/a	n/a	n/a
**Sex**	n/a	Any	n/a	n/a	n/a	n/a
**Sample size for primary and secondary outcomes**	**Density of adult anophelines:** 16 houses	**Anaemia prevalence in children:** 755 children in 500 houses. **Density of adult anophelines:** 500 houses (100 per year in the full screening arm, 100 per year in the ceiling arm, 50 per year in the control arm)	**Indoor density of adult anophelines:** 40 households (20 in the screened arm and 20 in the control arm)	**Density of adult anophelines:** 321 houses (231 in treatment arm and 90 in control arm)	**Density of adult anophelines:** 12 houses	**Density of adult anophelines:** 4 local houses (one block) and 4 experimental huts (one block)
**Intervention**	Screening of gables ends and eaves	House screening	House screening	House screening with repellent plants	Screening of eaves	House screening
**Details of the intervention**	Four experimental rounds: one house randomly assigned control and three houses assigned screening of gables with old bednets, untreated shade cloth or deltamethrin–impregnated shade cloth for two weeks; followed by screening gables and eaves for one week.	Houses were randomised to one of three arms: (1) no screening, (2) screened ceilings or (3) full screening (screened doors, windows and closed eaves using a mixture of sand, rubble and cement as is normal local practice).	Doors and windows were screened with metal mesh and eaves closed with mud.	Screening of house entry points by planting the densely foliated repellent plant *Lantana camara* around houses	In the first of two four–week intervention periods, 6 of 12 houses (with no windows and screened doors) were randomly selected and eaves blocked. Before the second period, the 6 homes with blocked eaves had them opened, and the 6 homes with open eaves had them closed.	Four repetitions of four experimental treatments over four nights in each block. No screening on the first night; on the subsequent three nights three of the four houses in each block had three identical treatments, changed each night (screening the eaves, windows and then doors).
**Duration of intervention**	3 weeks	6 months in each study year	Two months	10 months	4 weeks	16 days
**Co–interventions**	None	None. 30% slept under ITNs	Untreated bednets	None	Door screening	Untreated bednet
**Co–interventions equal?**	n/a	n/a	Yes	n/a	Yes	Yes
**Outcomes included in the review**	**1. Density of adult anophelines,** measured by nightly CDC light trap collections.	**1. Anaemia prevalence in children: Hb density/house in children,** measured by cross–sectional surveys pre– and post–intervention. **2. Density of adult mosquitoes**, measured by fortnightly CDC light trap collections in all houses. **3. Parasite prevalence**, measured by RDTs in pre/post cross–sectional surveys.	**1. Density of adult** anophelines, measured by CDC light trap collections on four consecutive nights every second week in all households over two months.	**'1. Density of adult anophelines** ,measured by ≈8 CDC light trap collections across all houses per week.	**1. Density of adult anophelines**, sampled using CDC light trap collections one night every two weeks.	**1. Density of adult anophelines** ,measured by nightly CDC light trap collections.
**Continent**	Africa	Africa	Africa	Africa	Africa	Africa
**Country**	Mozambique	The Gambia	Ethiopia	Tanzania	The Gambia	Tanzania
**Urban or rural**	Rural	Rural	Rural	Rural	Rural	Rural
**Primary vectors**	*An. funestus, An. gambiae* s.l	*An. gambiae s.s., Anopheles arabiensis*	*An. arabiensis*	*An. gambiae s.l; An. funestus s.s.*	*An. gambiae s.s., Anopheles arabiensis*	*An. gambiae s.l; An. funestus s.s.*
**Transmission**	Not reported	Moderate	Moderate	High	Moderate	High
**Funding**	Bill and Melinda Gates Foundation	Medical Research Council (UK)	Centre for International Health, University of Bergen in Norway; Arba Minch University	Xerox Foundation	Medical Research Council (UK)	Valent Bioscience Corporation, CDC, USAID, Addessium Foundation, Wellcome Trust

Pilot study: study with less than one year or transmission season of baseline data and/or intervention implemented for less than one year or transmission season; CDC: Centers for Disease Control and Prevention; OR: Odds Ratio; IRR: Incidence Rate Ratio; ITN: Insecticide-treated net.

Tusting LS, Ippolito M, et al. The evidence for improving housing to reduce malaria: a systematic review and meta-analysis.

b. Characteristics of observational studies included in the quantitative analysis (n=66)

Study	Country	Setting	Primary vectors	Transmission	LLIN coverage	IRS coverage	Study design	Study size	Age group	Recruitment of participants	Control group	Follow –up	Outcomes included
**Abe 2009 VNM**[7]	Vietnam	Rural	*An. dirus*	Low	50.4% coverage (any net)	Not reported	Cross–sectional	682	All ages	Community: All inhabitants of one village were surveyed	n/a	n/a	Malaria infection (microscopy)
**Adiamah 1993 GMB**[8]	The Gambia	Peri–urban	*An. gambiase s.l.*	Moderate	29% coverage (any bednet)	Not reported	Case–control	253	<10 yrs	Health facility: Children with mild malaria attending district hospital, age–matched as controls for children with severe malaria	Community controls: healthy, resident >500m from the cases, age–matched	n/a	Mild malaria (parasitaemia plus fever, microscopy); density of adult Anophelines (geometric mean number adult Anopheles per light trap catch)
**Al–Mekhlafi 2011 YEM**[9]	Yemen	Rural	*An. arabiensis*, *An. culicifacies*	Low	Not reported overall	Not reported overall	Cross–sectional	287	All ages	Health facility: febrile patients presenting	n/a	n/a	Malaria infection (microscopy)
**Al–Taiar 2009 YEM**[10]	Yemen	Rural	*An. arabiensis*	Low	8% coverage in study children (any net)	Not reported overall	Case–control	628	6m – 10 yrs	Health facility: Recruited consecutively from health centres; only one child per family was recruited	Community controls: age– and area of residence matched, healthy (no malaria infection or history of malaria in past 6 months), selected randomly from same community	n/a	Clinical malaria (microscopy)
**Animut 2013**[11]	Ethiopia	Rural	*An. arabiensis*	Not reported	Not reported	Not reported	Cohort	Pyrethrum spray catch: 10 randomly selected houses on 2 nights per village per month	n/a	Randomly sampled from 3 villages	n/a	24 months	Density of *An. arabiensis* (mean number *An. arabiensis* per pyrethrum spray catch)
**Asante 2013 GHA**[12]	Ghana	Urban and rural	Not stated	High	47%	Not reported	Cohort	1855	0–12 months	Health facilities: infants born to all mothers with and without placental malaria resident in 42 communities	n/a	12 months	Clinical malaria (RDT and microscopy; ACD through monthly home visits and PCD at clinics)
**Barber 1935 GRC**[13]	Greece	Rural	*An. elutus*, *An.maculipennis*	Low	Not reported	Not reported	Cross–sectional (clinical data); cohort (entomological data)	461 houses	1–11 months	All children aged 1–11 months resident in five villages (clinical data); all households in five villages (entomological data)	n/a	18 months	Malaria infection (microscopy); density of adult anophelines (mean number An. elutus and An. maculipennis per resting catch)

b. Characteristics of observational studies included in the quantitative analysis (n=66) (continued)

Study	Country	Setting	Primary vectors	Transmission	LLIN coverage	IRS coverage	Study design	Study size	Age group	Recruitment of participants	Control group	Follow –up	Outcomes included
**Bosman 1992**[14]	Republic of Guinea	Urban and rural	*An. gambiae s.l.*	High	Not reported	Not reported	Cross–sectional	44 pyrethrum spray catches in three villages	n/a	Not reported	n/a	n/a	Density of adult Anophelines (August monthly mean number adult Anopheles per pyrethrum spray catch)
**Bradley 2013 GNQ**[15]	Bioko, Equatorial Guinea	Rural, coastal	*An. funestus, An. gambiae*	High	5%	Not reported	Cross–sectional (repeat surveys)	22726	2–14 years	Randomly sampled from census survey	n/a	n/a	Malaria infection (RDT)
**Briggs–Watson 1940 USA**[16]	USA	Rural	*An.quadrimaculatus*	Low	Not reported	Not reported	Cross–sectional	1118 individuals (143 individuals)	All ages	Purposefully selected from community	n/a	n/a	Malaria infection (microscopy)
**Brooker 2004 KEN**[17]	Kenya	Rural, highland	*An. gambiase s.l.*	Low	3%	Not reported	Case–control	284	7–18 years (school age)	Recruited using active case detection from three schools over 10 weeks	Age– and school–matched children with absence of symptoms or no parasitaemia	n/a	Clinical malaria (microscopy)
**Burkot 1989**[18]	Papua New Guinea	Rural	*An. punctulatus*	High	88% reported sleeping under any net	Not reported	Cross–sectional	195 houses	n/a	Households selected from eight villages (not stated how)	n/a	12 months	Human biting rate (mean number of Anopheles per human landing catch)
**Butraporn 1986 THA**[19]	Thailand	Rural	*Not stated*	High	60% report regular use of bednet	Not reported	Case–control	698	All ages	Systematically sampled from list of malaria–positive cases residing in nine villages	Community controls (selection not described), matched on age, sex, village of residence	n/a	Malaria infection
**Charlwood 2003**[20]	Sao Tome	Peri–urban	*An. gambiae*	Not reported	Not reported	Not reported	Cross–sectional	22 landing catches in homes at ground level; 8 inside houses built on stilts	n/a	Not stated	n/a	n/a	Human biting rate (mean number of Anopheles per man hour of collection (HLC))
**Coleman 2010 ZAF**[21]	South Africa	Urban	*An. arabiensis*	Low	17% report sleeping under any net	68%	Case–control	212 households	All ages	Households with at least one confirmed malaria case in study area within study period	Community controls (three nearest households to case households with no confirmed malaria during the same period)	n/a	At least one confirmed case of clinical malaria in household

b. Characteristics of observational studies included in the quantitative analysis (n=66) (continued)

Study	Country	Setting	Primary vectors	Transmission	LLIN coverage	IRS coverage	Study design	Study size	Age group	Recruitment of participants	Control group	Follow –up	Outcomes included
**Coogle 1927**[22]	USA	Rural	*An. quadrimaculatus*	Low	Not reported	Not reported	Cohort	208 (estimated)	n/a	Purposefully selected from the community	n/a	Not reporte d	Density of adult anophelines (mean number *An. quadrimaculatus* per home
**Dahesh 2009 EGY**[23]	Egypt	Rural, lowland	*Not stated*	Not reported	Not reported	Not reported	Cohort	333	All ages	All inhabitants of one village	n/a	n/a	Malaria infection
**Danis–Lozano 2007 MEX**[24]	Mexico	Rural	*An. pseudopunctipennis; An. albimanus.*	Low	54% study participants reported always sleeping under net	Not reported	Case–control	357	All ages	Recruited from 60 villages by active case detection and by passive case detection at health facilities from community health workers or health clinics	Community controls, age–matched to within 5 years, resident in the study area, with no parasitaemia and no antibodies to P. vivax	n/a	Clinical malaria (microscopy)
**de Almeida 2010 TLS**[25]	East Timor	Rural	*Not stated*	Low	49% study participants reported sleeping under a bednet	Not reported	Cross–sectional	216	All ages	All inhabitants of 71 households (selection not described) were sampled	n/a	n/a	Malaria infection (microscopy)
**De Beaudrap 2011 UGA**[26]	Uganda	Urban and rural	*Not stated*	Moderate	45–65% households reported using at least one net	Not reported	Cross–sectional	1325	0–5 years	20 children randomly selected from each of 33 villages	n/a	n/a	Malaria infection (microscopy and RDT)
**Ekpenyong 2008 NGA**[27]	Nigeria	Rural	*Not stated*	High	3% reported sleeping under bednet	Not reported	Cross–sectional	1296	4–15 years	36 children randomly selected from six randomly selected schools every month for six months	n/a	n/a	Malaria infection (microscopy)
**Ernst 2009 KEN**[28]	Kenya	Rural, highland	*An. gambiae s.s.*	Low	4% reported sleeping under bednet	Not reported	Case–control	1468	All ages	Individuals presenting to two health centres with malaria symptoms and positive blood smear	Community controls: selected from census, matched by area of residence and age category, with no malaria symptoms the previous month and no history of confirmed malaria in the study period	n/a	Clinical malaria (microscopy)

b. Characteristics of observational studies included in the quantitative analysis (n=66) (continued)

Study	Country	Setting	Primary vectors	Transmission	LLIN coverage	IRS coverage	Study design	Study size	Age group	Recruitment of participants	Control group	Follow –up	Outcomes included
**Gamage–Mendis 1991[29]**	Sri Lanka	Rural	*An. subpictus, An. culicifacies*	Moderate	7% use of bednets	Not reported	Cohort	279 PSC in 146 houses	n/a	Randomly selected from one village	n/a	12 months	Indoor resting density (geometric mean number of *Anopheles* per trap per night)
**Geissbuhler 2007[30]**	Tanzania	Urban and rural	*An. gambiae* s.s., *An. arabiensis, An. merus*	Moderate	83% reported sleeping under a net	Not reported	Cross–sectional	216 houses (1 night long HLC in each)	n/a	Selected from households enrolled as sentinel sites for UMCP	n/a	10 weeks	Human biting rate (mean number of bites received by those sleeping indoors (HLC))
**Ghebreyseus 2000 ETH[31]**	Ethiopia	Rural, highland	*An.arabiensis*	Low	Not reported	Not reported	Cohort	2114	0–10 years	All children aged 0–10 years resident in six villages	n/a	12 months	Clinical malaria (microscopy)
**Guthman 2001 PER[32]**	Peru	Rural	*An. albimanus; An. pseudopunctipennis; An. calderoni*	Low	35% reported sleeping under bednet the previous night	3% in past 6 months	Case–control	1292	All ages	All individuals with malaria symptoms and malaria infection within a community, detected by active case detection	Community controls: age–, sex– and village–matched, with no malaria infection or history of malaria in past 28 days	n/a	Clinical malaria (microscopy)
**Hagmann 2003 STP[33]**	Principe	Urban and rural	*An. gambiae*	Moderate	54% reported using bednets		Cross–sectional	1062	All ages	All inhabitants of six communities	n/a	n/a	Malaria infection (microscopy)
**Haque 2013 BGD[34]**	Bangladesh	Rural	*An. baimai; An. minimus s.l.; An. annularis*	High	71% households owned at least 0.5 nets per person	Not reported	Cohort	1634 (households)	Households	All households in all 54 villages in one administrative area	n/a	24 months	At least one malaria case per household detected by passive case detection at health facilities
**Hiscox 2013[35]**	Lao PDR	Rural	*An. philippinensis, An. nivipes, An. aconitus*	Not reported	Not reported	Not reported	Cross–sectional	192 households (96 modern; 96 traditional)	n/a	Randomly selected from census of all households in study area	n/a	n/a	Human biting rate (mean number of *Anopheles* per CDC light trap per night)
**Hustache 2007 GUF[36]**	French Guiana	Rural, forest	*An. darlingi*	Moderate	70% children reported sleeping under bednets	Not reported	Cohort	369	0–5 years	All children aged 0–5 years in one village	n/a	12 months	Clinical malaria (microscopy)

b. Characteristics of observational studies included in the quantitative analysis (n=66) (continued)

Study	Country	Setting	Primary vectors	Transmission	LLIN coverage	IRS coverage	Study design	Study size	Age group	Recruitment of participants	Control group	Follow –up	Outcomes included
**Kaur 2009 MYS**[37]	Malaysia	Rural, forest	*An. maculatus*	Moderate	95% people reported always sleeping under ITN	91% in the past yer	Cross–sectional	520	All ages	Residents of 10 villages randomly selected from 19 villages in one district	n/a	n/a	Malaria infection (microscopy)
**Kirby 2008**[38]	The Gambia	Rural	*An. gambiae* s.s., *An. arabiensis, An. melas*	Moderate	Not reported	Not reported	Cross–sectional	976 houses (with sentinel LTC in 4 additional houses to adjust for nightly density variation)	n/a	Randomly selected from 46 residential DSS blocks in town and 22 satellite villages	n/a	n/a	Human biting rate (mean number of *Anopheles* per CDC light trap per night)
**Konradsen 2003 LKA**[39]	Sri Lanka	Rural	*An. culicifacies, An. subpictus*	Not reported	Very low'	Aprox 82% 'good' coverage	Cohort	2640 collections in 473 houses (indoor resting densities sampled fortnightly)	n/a	New 10% sample of houses from 7 contiguous villages randomly selected every fortnight	n/a	30 months	Presence (vs absence) of *An. subpictus* in each collection (pyrethrum spray catch)
**Koram 1995 GMB**[40]	The Gambia	Peri–urban	*An. gambiae s.s.*	Moderate	32% reported using bednets	Not reported	Case–control	768	3m to 10 years	Children presenting to clinic with mild malaria	Community controls: age–matched, randomly selected from compound at least 400m from case compound	n/a	Clinical malaria (microscopy)
**Kreuels 2008 GHA**[41]	Ghana	Rural	*An.gambiae s.s., An. arabiensis, An.funestus*	High	31% reported using bednet	Not reported	Cohort	535	3 months to 2 years	Participants enrolled into a randomized, double–blind, placebo–controlled study of IPTi	n/a	21 months	Clinical malaria (microscopy)
**Lindsay 1988**[42]	The Gambia	Rural	*An. gambiae* s.s., *An. arabiensis*	Not reported	Not reported	Not reported	Cohort	Not reported	n/a	Selected from 4 hamlets	n/a	6 months	Density of adult Anophelines (mean number of *Anopheles* per night)
**Lindsay 1995**[43]	The Gambia	Rural	*An. gambiae* s.s., *An. arabiensis*	High	90% (children, any net)	Not reported	Cohort	381 bednets in use in one village	n/a	140 bednets sampled randomly every three weeks from bednets of all 381 children aged 0–7 years in one village	n/a	18 months	Density of of adult anophelines (mean number *An. gambiae* s.l. caught under bednets)

b. Characteristics of observational studies included in the quantitative analysis (n=66) (continued)

Study	Country	Setting	Primary vectors	Transmission	LLIN coverage	IRS coverage	Study design	Study size	Age group	Recruitment of participants	Control group	Follow –up	Outcomes included
**Liu 2014 TZA**[44]	Tanzania	Rural	*An. gambiae s.l.; An. arabiensis*	Moderate	57% household ownership	Not reported	Cohort	435 houses	2 months – 2 years	Randomly selected from participants of IPTi trial	n/a	22 months	Clinical malaria (microscopy); density of adult anophelines (mean number of *Anopheles* collected per household)
**Magalhaes 2012 AGO**[45]	Angola	Rural	*Not stated*	Moderate	28% mothers owned bednets	Not reported	Cross–sectional	2265	≤15 years	Households randomly selected from DSS administrative area	n/a	n/a	Malaria infection (RDT)
**Maheu–Giroux 2010 PER**[46]	Peru	Rural, forest	*An. darlingi*	Low	Not reported	Not reported	Cohort	1018	All ages	All households within catchment of health facility sampled, 90% consented to provide data	n/a	30 months	Incidence of clinical malaria (microscopy, retrospective passive case detection)
**Malik 2003 SDN**[47]	Sudan	Urban	*An. arabiensis*	Moderate	11% households used bednets	Not reported	Cross–sectional	8092	All ages	Households randomly selected from three administrative areas and surveyed in three surveys	n/a	n/a	Malaria infection (microscopy)
**Mmbando 2011 TZA**[48]	Tanzania	Rural	*Not stated*	High	'High'	Not reported	Cross–sectional	12298	0–19 years	Randomly selected from 14 villages	n/a	n/a	Malaria infection (microscopy)
**Mutuku 2011**[49]	Kenya	Rural	*An. funestus, An. gambiae* s.s., *An. arabiensis*	Moderate	68% coverage of all sleeping places (any net)	Not reported	Cohort	1777 collections in 20 houses sampled weekly	n/a	Randomly selected from two villages	n/a	89 weeks	Density of adult Anophelines (pyrethrum spray catch)
**Nahum 2010 BEN**[50]	Benin	Peri–urban	*An. gambiae* s.s.; *An. melas*	Moderate, seasonal	55% children slept under a bednet	Not reported	Cohort	553	6 months to 5 years	All eligible children from households enumerated in census survey	n/a	18 months	Clinical malaria (microscopy)
**Oesterholt 2006 TZA**[51]	Tanzania	Rural	*An. arabiensis*	Low	Not reported	Not reported	Cohort	3388	All ages	All inhabitants of one village included; cases identified by passive case detection at clinic	n/a	12 months	Clinical malaria (fever plus parasitaemia, microscopy)

b. Characteristics of observational studies included in the quantitative analysis (n=66) (continued)

Study	Country	Setting	Primary vectors	Transmission	LLIN coverage	IRS coverage	Study design	Study size	Age group	Recruitment of participants	Control group	Follow –up	Outcomes included
**Ong'Echa 2006 KEN[52]**	Kenya	Rural	*An. gambiae s.s.; An. arabiensis; An.funestus*	High	35% households reported using bednets	Not reported	Case–control	374	0–3 years	Children attending district hospital with malaria symptoms and positive smear	Hospital controls: recruited from MCH clinic at the same hospital during presentation for EPI vaccinations, malaria–negative smear and no history of fever or diarrhoea in past 14 days	n/a	Malaria anaemia, malaria infection
**Osterbauer 2012 UGA[53]**	Uganda	Rural	*Not stated*	High	34% study participants reported sleeping under ITN the previous night	None	Cross–sectional	600	4–6 months	Recruited using convenience sampling at antenatal clinic in district hospital	n/a	n/a	Malaria infection (microscopy)
**Ouma 2007 KEN[54]**	Kenya	Urban and peri–urban	*Not stated*	High	32% women used ITN in pregnancy	Not reported	Cross–sectional	685	15–45 years	First ANC attenders at district hospital screened for folic acid supplementation trial	n/a	n/a	Malaria infection (microscopy)
**Pardo 2006 GNQ[55]**	Bioko, Equatorial Guinea	Urban and rural	*An. gambiae s.s.; An. funestus*	High	38% slept under any net	78%	Cross–sectional	433	0–5 years	Randomly selected from the community	n/a	n/a	Malaria infection (microscopy)
**Peterson 2009a ETH[56]**	Ethiopia	Peri–urban	*An. arabiensis*	Low	3% households owned a ITN at baseline	Not reported	Cohort	294 (households; with 1367 individuals)	>1 year	Random sampling of every fourth house in city administrative unit	n/a	4 months	Clinical malaria (microscopy)
**Peterson 2009b ETH[57]**	Ethiopia	Peri–urban	*An. arabiensis*	Low	4% compounds owned an ITN	Not reported	Cohort	1187 (compounds; with 8008 individuals)	>1 year	All compounds within one city administrative unit	n/a	4 months	Clinical malaria (microscopy)
**Rulisa 2013 RWA[58]**	Rwanda	Rural	*Not stated*	Moderate	97% people reported sleeping under bednet	94% (self reported coverage)	Cross–sectional	520 (households; with 2634 individuals)	All ages	Households in which one member presented at study health facility with fever or history of fever	n/a	n/a	At least one malaria infection in household (microscopy)
**Russell 2013[59]**	Tanzania	Rural	*An. arabiensis, An. gambiae s.s*	High	Number of bednets in use per person ranged from 0.44 to 0.63 in high density clusters	Not reported	Cohort	72 houses sampled monthly	n/a	Randomly selected from two villages	n/a	12 months	Density of adult *An. gambiae* s.l. (CDC light trap))

b. Characteristics of observational studies included in the quantitative analysis (n=66) (continued)

Study	Country	Setting	Primary vectors	Transmission	LLIN coverage	IRS coverage	Study design	Study size	Age group	Recruitment of participants	Control group	Follow –up	Outcomes included
**Sintasath 2005 ERI[60]**	Eritrea	Rural; highland and lowland	*An. arabiensis*	Low	Not reported	39% villages were covered	Cross–sectional	2779 households	All ages	Randomly selected from villages in six zobas that were selected due to greater ecological diversity and population density	n/a	n/a	At least one malaria infection in household (RDT)
**Siri 2010 KEN[61]**	Kenya	Urban to semi–rural	*Not stated*	High	46% children slept under bednet the previous week	Not reported	Case–control	906	0–7 years	Children admitted to district hospital inpatient ward with malaria anaemia and high parasitaemia	Community controls: Healthy respondents to a concurrent citywide knowledge, attitude, and practice survey; aged 0–7 years	n/a	Malaria anaemia (microscopy)
**Temu 2012 MOZ[63]**	Mozambique	Rural	*An. gambiae, An. funestus*	High	23% children slept under ITN	65% children lived in homes with IRS	Cross–sectional	8338	1–15 years	Surveyed as part of a Malaria Indicator Survey at 19 sentinel sites	n/a	n/a	Malaria infection (RDT)
**Townes 2013 MWI[64]**	Malawi	Rural	*An. funestus, An. gambiae s.s., An. arabiensis*	High	53% households at least one bednet	Not reported	Cross–sectional	390	4 months to 5 years	All children aged 4 months to 5 years resident in 10 randomly selected villages	n/a	n/a	Malaria infection (RDT)
**Van der Hoek 2003 LKA[65]**	Sri Lanka	Rural	*An. culicifacies*	Low	6% participants reported sleeping under a bednet in the past 2 weeks	None	Case–control	875	All ages	Inhabitants of seven villages who attended district hospital or mobile clinic with positive bloor smear	Community controls: randomly selected from census of same villages who did not report a malaria episode in previous two weeks	n/a	Malaria infection (microscopy)
**Wanzirah 2015[66] (included as unpublished data at the time of the search and since published)**	**Uganda**	Rural	*An. gambiae s.s., An. arabiensis*	Moderate to high	99% reported sleeping under any net the previous night	None	Cohort	300 households	n/a	100 households randomly selected from census population in each of three sites	n/a	24 months	Clinical malaria (microscopy); malaria infection (microscopy); human biting rate (mean number of *Anopheles* per household per night)
**Winskill 2011 TZA[67]**	**Tanzania**	Rural	An. gambiae s.l.	High	46% slept under ITN	Not reported	Cross–sectional	1438	6 months – 13 years	All eligible residents of five villages	n/a	n/a	Malaria infection (microscopy)

b. Characteristics of observational studies included in the quantitative analysis (n=66) (continued)

Study	Country	Setting	Primary vectors	Transmission	LLIN coverage	IRS coverage	Study design	Study size	Age group	Recruitment of participants	Control group	Follow –up	Outcomes included
**Wolff 2001 MWI**[68]	Malawi	Rural	*Not stated*	Moderate	Not reported	Not reported	Cross–sectional	318	0–5 years	Residents of randomly selected houses built by Habitat for Humanity International and the closest traditional house to each	n/a	n/a	Malaria infection (microscopy)
**Woyessa 2013 ETH**[69]	Ethiopia	Rural	*Not stated*	Low	29% households own at least one ITN	Some coverage	Cross–sectional	3398	All ages	Households randomly selected from six administrative areas	n/a	n/a	Malaria infection (microscopy)
**Yamamoto 2010 BFA**[70]	Burkina Faso	Peri–urban	*Not stated*	High	49% participants resided in households reporting use of ITNs	Not reported	Case–control	283	0–9 years	Children presenting to district health facility with fever and parasitaemia	Community controls: age, sex, ethnicity and residence matched, selected from DSS database	n/a	Clinical malaria (microscopy)
**Ye 2006 BFA**[71]	Burkina Faso	Rural, peri–urban	*Not stated*	High	53% reported use of any net	Not reported	Cross–sectional	661	6 months – 5 years	Randomly selected using cluster sampling from four DSS sites	n/a	n/a	Malaria infection (microscopy)
**Yukich 2013 ETH**[72]	Ethiopia	Urban and rural	*An. arabiensis*	Low–moderate	42% participants lived in homes owning at least one ITN, 19% used a ITN the previous night	Not reported	Case–control	560	≥18 years	Individuals presenting at local health facility with fever and malaria infection	Individuals presenting at same health facility without malaria infection	n/a	Malaria infection (microscopy and RDT)
**Zhou 2007**[73]	Kenya	Rural, highland	*An. gambiae* s.s., *An. funestus*	High	Not reported	Not reported	Cohort	871 houses	n/a	Households randomly selected from two areas of 3x3km and 4x4km	n/a	9 months	Density of adult anophelines (mean number of *An. gambiae* s.s. per house (pyrethrum spray catch))

CDC: Centers for Disease Control and Prevention; OR: Odds Ratio; IRR: Incidence Rate Ratio; RDT: Rapid diagnostic test; ITN: Insecticide-treated net; LLIN: Long-lasting insecticidal net; IRS: Indoor residual spraying; PCD: Passive case detection; ACD: Active case detection; SES: socioeconomic status.

Tusting LS, Ippolito M, et al. The evidence for improving housing to reduce malaria: a systematic review and meta-analysis.

c. Characteristics of observational studies excluded from the quantitative analysis (n=18)

Study	Country	Setting	Primary vectors	Transmission	LLIN coverage	IRS coverage	Study design	Study size	Age group	Recruitment of participants	Control group	Follow – up	Reason for exclusion from quantitative analysis
**Ayele 2012 ETH**[74]	Ethiopia	Rural, highland	*Not stated*	Low	Not reported	Not reported	Cross–sectional	5708 households	Not stated	224 clusters of 25 households randomly selected from census	n/a	n/a	Confidence intervals cannot be replicated; data reporting not accurate
**Ayele 2013 ETH**[75]	Ethiopia	Rural, highland	*Not stated*	Low	Not reported	Not reported	Cross–sectional	5708 households	Not stated	224 clusters of 25 households randomly selected from census	n/a	n/a	Confidence intervals cannot be replicated; data reporting not accurate
**Bell 1997**[76]	Solomon Islands	Urban, coastal	*An. farauti s.l., An. punctulatus, An. koliensis*	Moderate	29.5% (any net); 12.7% (ITN)	Not reported	Cross–sectional	309	>=16 years	Every second outpatient from study health facilitites recruited if consent given and aged at least 16 yrs	n/a	n/a	No confidence intervals given; direction of comparison unclear
**Cano 2006**[77]	Equatorial Guinea	Rural	*An. gambiae* s.s., *An. moucheti, An. carnevalei*	High	31% slept under bednet (any)	Not reported	Cohort	One village with 37 households, each of which was surveyed 6–8 times over the collection period	n/a	Two sentinel houses were purposefully selected; 5 light traps were circulated among the remaining households	n/a	42 days	Regression coefficients given only
**de Barros 2011**[78]	Brazil	Rural	*An. darlingi*	Not reported	4% families reported sleeping under nets	Not reported	Cohort	333	All ages	All residents of a side road	n/a	30 months	Regression coefficients only
**Kibret 2010**[79]	Ethiopia	Rural, semi–arid	*An. arabiensis, An. pharoensis*	Moderate	Not reported	Not reported	Cross–sectional	2435	All ages	Households randomly selected from two villages	n/a	n/a	Regression coefficients only
**Lwetoijera 2013 TZA**[80]	Tanzania	Rural	*An. gambiae* s.l., *An.funestus*	Not reported	100% housheolds owned treated nets, 32% households owned mainly ITNs	Not reported	Cohort	72 randomly selected houses each month	n/a	Randomly selected from two villages each month	n/a	48 months	Regression coefficients given only
**Mala 2011**[81]	Kenya	Rural	*An. arabiensis*	Not reported	Not reported	Not reported	Cohort	20 houses	n/a	10 houses randomly selected from each of two sites	n/a	22 months	Regression coefficients given only
**Manah 2012**[82, 83]	Malaysia	Rural	*Not stated*	Low	Not reported	Not reported	Case–control	332	All ages	All malaria cases notified to the district health office within study period	Age– and sex–matched controls (no further details given)	n/a	House structure described as a risk factor for malaria infection but no data given
**Muturi 2008**	Kenya	Rural	*An. arabiensis, An. pharoensis*	Not reported	Not reported	Not reported	Cohort	30 houses	n/a	Randomly selected from 3 study sites	n/a	12 months	Regression coefficients given only
**Nkuo–Akenji 2006**[84]	Cameroon	Rural	*An. gambiae; A. funestus*	High	Not reported	Not reported	Cross–sectional	208	0–14 years	Selected from community (not stated how)	n/a	n/a	Confidence intervals not reported

c. Characteristics of observational studies excluded from the quantitative analysis (n=18) (continued)

Study	Country	Setting	Primary vectors	Transmission	LLIN coverage	IRS coverage	Study design	Study size	Age group	Recruitment of participants	Control group	Follow – up	Reason for exclusion from quantitative analysis
**Palsson 2004[85]**	Guinea Bissau	Peri–urban	*An. gambiae* s.l., *An. squamosus*	Moderate	94% people reported sleeping under nets (any)	Not reported	Cohort	30 houses sampled three times during each rainy season	n/a	10 houses selected from each of three areas (not stated how)	n/a	26 months (rainy season only)	Regression coefficients given only
**Somi 2007[86]**	Tanzania	Rural	*Not stated*	High	50% people reported sleeping under any net, 18% under an ITN	Not reported	Cross–sectional	2318 (household; with 7657 individuals)	All ages	Households randomly selected from two DSS sites	n/a	n/a	Regression coefficients only
**Somi 2008[87]**	Tanzania	Rural	*Not stated*	High	79% people reported sleeping under any net, 27% under an ITN	Not reported	Cross–sectional	557 (households; with 2034 individuals)	All ages	Households randomly selected from two DSS sites	n/a	n/a	Regression coefficients only
**Subramanian 1991[88]**	India	Rural	*An. fluviatilis*	Moderate	Not reported	Not reported	Cohort	1461	All ages	All inhabitants of one village	n/a	12 months	Insufficient data reported
**Sur 2006 IND[62]**	India	Urban	*An. stephensi*	Low	Very low	None	Cohort	60452	All ages	Individuals reporting to a study health post with fever were screened; denominator was total population from census	None	12 months	Relative risk reported only
**Tilaye 2007 ETH[89]**	Ethiopia	Urban	*Not reported*	Not reported	38% individuals belonged to households owning at least one bednet	18% individuals lived in homes with IRS in preceding year	Cross–sectional	734	All ages	Residents of households selected by multi–stage cluster sampling from three randomly selected administrative areas	n/a	n/a	House features included do not represent a comparison of modern versu traditional
**Van der Hoek 1998 LKA[90]**	Sri Lanka	Rural	*An. culicifacies*	Moderate	9% (any net)	90%	Cohort	280	All ages	All inhabitants of one village	n/a	12 months	Relative risk reported only

ITN: Insecticide-treated net; LLIN: Long-lasting insecticidal net; IRS: Indoor residual spraying; PCD: Passive case detection; ACD: Active case detection; SES: socioeconomic status; DSS: Demographic Surveillance System

References

1. Kampango A, Braganca M, Sousa BD, Charlwood JD. Netting barriers to prevent mosquito entry into houses in southern Mozambique: A pilot study. *Malar J* 2013; **12:** 1.

2. Kirby MJ, Ameh D, Bottomley C, et al. Effect of two different house screening interventions on exposure to malaria vectors and on anaemia in children in The Gambia: a randomised controlled trial. *Lancet* 2009; **374**: 998–1009.

3. Massebo F, Lindtjorn B. The effect of screening doors and windows on indoor density of *Anopheles arabiensis* in south–west Ethiopia: A randomized trial. *Malar J* 2013; **12:** 1.

4. Mng'ong'o FC, Sambali JJ, Sabas E, et al. Repellent plants provide affordable natural screening to prevent mosquito house entry in tropical rural settings–results from a pilot efficacy study. *PLoS ONE* 2011; **6:** 10.

5. Kirby MJ, Njie M, Dilger E, Lindsay SW. Importance of eaves to house entry by anopheline, but not culicine, mosquitoes. *J Med Entomol* 2009; **46**: 505–10.

6. Ogoma SB, Lweitoijera DW, Ngonyani H, et al. Screening mosquito house entry points as a potential method for integrated control of endophagic filariasis, arbovirus and malaria vectors. *PLoS Negl Trop Dis* 2010; **4:** 8.

7. Abe T, Honda S, Nakazawa S, et al. Risk factors for malaria infection among ethnic minorities in Binh Phuoc, Vietnam. *Southeast Asian J Trop Med Public Health* 2009; **40**: 18–29.

8. Adiamah JH, Koram KA, Thomson MC, Lindsay SW, Todd J, Greenwood BM. Entomological risk factors for severe malaria in a peri–urban area of The Gambia. *Ann Trop Med Parasitol* 1993; **87**: 491–500.

9. Al–Mekhlafi AM, Al–Mekhlafi HM, Mahdy MA, Azazy AA, Fong MY. Human malaria in the highlands of Yemen. *Ann Trop Med Parasitol* 2011; **105:** 187–95.

10. Al–Taiar A, Assabri A, Al–Habori M, et al. Socioeconomic and environmental factors important for acquiring non–severe malaria in children in Yemen: a case–control study. *Trans R Soc Trop Med Hyg* 2009; **103**: 72–8.

11. Animut A, Balkew M, Lindtjorn B. Impact of housing condition on indoor–biting and indoor–resting Anopheles arabiensis density in a highland area, central Ethiopia. *Malar J* 2013; **12**: 393.

12. Asante KP, Owusu–Agyei S, Cairns M, et al. Placental malaria and the risk of malaria in infants in a high malaria transmission area in Ghana: A prospective cohort study. *J Infect Dis* 2013; **208**: 1504–13.

13. Barber M, Rice J. Malaria studies in Greece – The relation to housing to malaria in certain villages of East Macedonia. *Am J Hyg* 1935; **22**: 512–38.

14. Bosman A, Modiano D, Voglino MC, et al. Malaria transmission in a central area of Futa Djalon (Guinea): results of a parasitological survey during the 1989 rainy season. *Parassitologia* 1992; **34**: 135–42.

15. Bradley J, Rehman AM, Schwabe C, et al. Reduced prevalence of malaria infection in children living in houses with window screening or closed eaves on Bioko Island, Equatorial Guinea. *PLoS ONE* 2013; **8**: e80626.

16. Briggs–Watson R, Maher H. An evaluation of mosquito–proofing for malaria control based on one year's observations. *Am J Hyg* 1940; **34**: 86–94.

17. Brooker S, Clarke S, Njagi JK, et al. Spatial clustering of malaria and associated risk factors during an epidemic in a highland area of western Kenya. *Trop Med Int Health* 2004; **9**: 757–66.

18. Burkot TR, Dye C, Graves PM. An analysis of some factors determining the sporozoite rates, human blood indexes, and biting rates of members of the *Anopheles punctulatus* complex in Papua New Guinea. *Am J Trop Med Hyg* 1989; **40**: 229–34.

19. Butraporn P, Sornmani S, Hungsapruek T. Social, behavioural, housing factors and their interactive effects associated with malaria occurrence in east Thailand. *Southeast Asian J Trop Med Public Health* 1986; **17**: 386–92.

20. Charlwood JD, Pinto J, Ferrara PR, et al. Raised houses reduce mosquito bites. *Malar J* 2003; **2**: 45.

21. Coleman M, Coleman M, Mabaso ML, et al. Household and microeconomic factors associated with malaria in Mpumalanga, South Africa. *Trans R Soc Trop Med Hyg* 2010; **104**: 143–7.

22. Coogle C. Preliminary report of screening studies in Leflore County, Miss. *Pub Health Rep* 1927; **42**: 1101.

23. Dahesh SM, Bassiouny HK, El–Masry SA. Socioeconomic and environmental factors affecting malaria infection in Fayoum Governorate, Egypt. *J Egypt Soc Parasitol* 2009; **39**: 511–23.

24. Danis–Lozano R, Rodriguez MH, Betanzos–Reyes AF, et al. Individual risk factors for *Plasmodium vivax* infection in the residual malaria transmission focus of Oaxaca, Mexico. *Salud Publica de Mexico* 2007; **49**: 199–209.

25. de Almeida A, do Rosario VE, Arez AP, Cravo P. Malaria epidemiology in the Democratic Republic of East Timor. *Asian Pacific J Trop Med* 2010; **3**: 283–7.

26. De Beaudrap P, Nabasumba C, Grandesso F, et al. Heterogeneous decrease in malaria prevalence in children over a six–year period in south–western Uganda. *Malar J* 2011: **10**: 132.

27. Ekpenyong EA, Eyo JE. Malaria control and treatment strategies among school children in semi–urban tropical communities. *West Indian Med J* 2008; **57**: 456–61.

28. Ernst KC, Lindblade KA, Koech D, et al. Environmental, socio–demographic and behavioural determinants of malaria risk in the western Kenyan highlands: a case–control study. *Trop Med Int Health* 2009; **14**: 1258–65.

29. Gamage–Mendis AC, Carter R, Mendis C, De Zoysa APK, Herath PRJ, Mendis KN. Clustering of malaria infections within an endemic population: Risk of malaria associated with the type of housing construction. *Am J Trop Med Hyg* 1991; **45**: 77–85.

30. Geissbuhler Y, Chaki P, Emidi B, et al. Interdependence of domestic malaria prevention measures and mosquito–human interactions in urban Dar es Salaam, Tanzania. *Malar J* 2007; **6**: 126.

31. Ghebreyesus TA, Haile M, Witten KH, et al. Household risk factors for malaria among children in the Ethiopian highlands. *Trans R Soc Trop Med Hyg* 2000; **94**: 17–21.

32. Guthmann JP, Hall AJ, Jaffar S, Palacios A, Lines J, Llanos–Cuentas A. Environmental risk factors for clinical malaria: a case–control study in the Grau region of Peru. *Trans R Soc Trop Med Hyg* 2001; **95**: 577–83.

33. Hagmann R, Charlwood JD, Gil V, Ferreira C, do Rosario V, Smith TA. Malaria and its possible control on the island of Principe. *Malar J* 2003; **2**: 15.

34. Haque U, Glass GE, Bomblies A, et al. Risk factors associated with clinical malaria episodes in Bangladesh: a longitudinal study. *Am J Trop Med Hyg* 2013; **88**: 727–32.

35. Hiscox A, Khammanithong P, Kaul S, et al. Risk factors for mosquito house entry in the Lao PDR. *PLoS One* 2013; **8**: e62769.

36. Hustache S, Nacher M, Djossou F, Carme B. Malaria risk factors in Amerindian children in French Guiana. *Am J Trop Med Hyg* 2007; **76**: 619–25.

37. Kaur G. Predictors of malaria among Malaysian aborigines. *Asia–Pacific J Public Health / Asia–Pacific Academic Consortium for Public Health* 2009; **21:** 205–15.

38. Kirby MJ, Green C, Milligan PM, et al. Risk factors for house–entry by malaria vectors in a rural town and satellite villages in The Gambia. *Malar J* 2008; **7**: 2.

39. Konradsen F, Amerasinghe P, van der Hoek W, Amerasinghe F, Perera D, Piyaratne M. Strong association between house characteristics and malaria vectors in Sri Lanka. *Am J Trop Med Hyg* 2003; **68**: 177–81.

40. Koram KA, Bennett S, Adiamah JH, Greenwood BM. Socio–economic risk factors for malaria in a peri–urban area of The Gambia. *Trans R Soc Trop Med Hyg* 1995; **89**: 146–50.

41. Kreuels B, Kobbe R, Adjei S, et al. Spatial variation of malaria incidence in young children from a geographically homogeneous area with high endemicity. *J Infect Dis* 2008; **197**: 85–93.

42. Lindsay SW, Snow RW. The trouble with eaves; house entry by vectors of malaria. *Trans R Soc Trop Med Hyg* 1988; **82**: 645–6.

43. Lindsay SW, Armstrong Schellenberg JRM, Zeiler HA, Daly RJ, Salum FM, Wilkins HA. Exposure of Gambian children to *Anopheles gambiae* malaria vectors in an irrigated rice production area. *Med Vet Entomol* 1995; **9**: 50–8.

44. Liu JX, Bousema T, Zelman B, et al. Is housing quality associated with malaria incidence among young children and mosquito vector numbers? Evidence from Korogwe, Tanzania *PLoS ONE* 2014; **9:** e87358.

45. Magalhaes RJ, Langa A, Sousa–Figueiredo JC, Clements AC, Nery SV. Finding malaria hot–spots in northern Angola: the role of individual, household and environmental factors within a meso–endemic area. *Malar J* 2012; **11**: 385.

46. Maheu–Giroux M, Casapia M, Soto–Calle VE, et al. Are fish farming activities contributing to malaria transmission in the Peruvian Amazon? *Am J Trop Med Hyg* 2009; **81**: 52–3.

47. Malik EM, Ahmed ES, Elkhalifa SM, Hussein MA, Sulieman AM. Stratification of Khartoum urban area by the risk of malaria transmission. *E Med Health J* 2003; **9**: 559–69.

48. Mmbando BP, Kamugisha ML, Lusingu JP, et al. Spatial variation and socio–economic determinants of *Plasmodium falciparum* infection in northeastern Tanzania. *Malar J* 2011; **10**: 145.

49. Mutuku FM, King CH, Mungai P, et al. Impact of insecticide–treated bed nets on malaria transmission indices on the south coast of Kenya. *Malar J* 2011; **10**: 356.

50. Nahum A, Erhart A, Maye A, et al. Malaria incidence and prevalence among children living in a peri–urban area on the coast of Benin, west Africa: a longitudinal study. *Am J Trop Med Hyg* 2010; **83**: 465–73.

51. Oesterholt MJ, Bousema JT, Mwerinde OK, et al. Spatial and temporal variation in malaria transmission in a low endemicity area in northern Tanzania. *Malar J* 2006; **5**: 98.

52. Ong'echa JM, Keller CC, Were T, et al. Parasitemia, anemia, and malarial anemia in infants and young children in a rural holoendemic *Plasmodium falciparum* transmission area. *Am J Trop Med Hyg* 2006; **74**: 376–85.

53. Osterbauer B, Kapisi J, Bigira V, et al. Factors associated with malaria parasitaemia, malnutrition, and anaemia among HIV–exposed and unexposed Ugandan infants: a cross–sectional survey. *Malar J* 2012; **11**: 432.

54. Ouma P, Eijk A, Hamel M, et al. Malaria and anaemia among pregnant women at first antenatal clinic visit in Kisumu, western Kenya. *Trop Med Int Health* 2007; **12**: 1515–23.

55. Pardo G, Descalzo MA, Molina L, et al. Impact of different strategies to control *Plasmodium* infection and anaemia on the island of Bioko (Equatorial Guinea). *Malar J* 2006; **5**: 10.

56. Peterson I, Borrell LN, El–Sadr W, Teklehaimanot A. Individual and household level factors associated with malaria incidence in a highland region of Ethiopia: a multilevel analysis. *Am J Trop Med Hyg* 2009; **80**: 103–11.

57. Peterson I, Borrell LN, El–Sadr W, Teklehaimanot A. A temporal–spatial analysis of malaria transmission in Adama, Ethiopia. *Am J Trop Med Hyg* 2009; **81**: 944–9.

58. Rulisa S, Kateera F, Bizimana JP, et al. Malaria prevalence, spatial clustering and risk factors in a low endemic area of Eastern Rwanda: a cross sectional study. *PLoS One* 2013; **8**: e69443.

59. Russell TL, Lwetoijera DW, Knols BG, Takken W, Killeen GF, Kelly–Hope LA. Geographic coincidence of increased malaria transmission hazard and vulnerability occurring at the periphery of two Tanzanian villages. *Malar J* 2013; **12**: 24.

60. Sintasath DM, Ghebremeskel T, Lynch M, et al. Malaria prevalence and associated risk factors in Eritrea. *Am J Trop Med Hyg* 2005; **72** 682–7.

61. Siri JG, Wilson ML, Murray S, et al. Significance of travel to rural areas as a risk factor for malarial anemia in an urban setting. *Am J Trop Med Hyg* 2010; **82**: 391–7.

62. Sur D, von Seidlein L, Manna B, et al. The malaria and typhoid fever burden in the slums of Kolkata, India: data from a prospective community–based study. *Trans R Soc Trop Med Hyg* 2006; **100**: 725–33.

63. Temu EA, Coleman M, Abilio AP, Kleinschmidt I. High prevalence of malaria in Zambezia, Mozambique: the protective effect of IRS versus increased risks due to pig–keeping and house construction. *PLoS One* 2012; **7**: e31409.

64. Townes LR, Mwandama D, Mathanga DP, Wilson ML. Elevated dry–season malaria prevalence associated with fine–scale spatial patterns of environmental risk: a case—control study of children in rural Malawi. *Malar J* 2013; **12**: 407.

65. Van Der Hoek W, Konradsen F, Amerasinghe PH, Perera D, Piyaratne MK, Amerasinghe FP. Towards a risk map of malaria for Sri Lanka: the importance of house location relative to vector breeding sites. *Int J Epidemiol* 2003; **32**: 280–5.

66. Wanzirah H, Tusting LS, Arinaitwe E, et al. Mind the gap: house structure and the risk of malaria in Uganda. *PLoS ONE* 2014; **10**:e0117396

67. Winskill P, Rowland M, Mtove G, Malima RC, Kirby MJ. Malaria risk factors in north–east Tanzania. *Malar J* 2011; **10**: 98.

68. Wolff CG, Schroeder DG, Young MW. Effect of improved housing on illness in children under 5 years old in northern Malawi: cross sectional study. *BMJ* 2001; **322**: 1209–12.

69. Woyessa A, Deressa W, Ali A, Lindtjorn B. Malaria risk factors in Butajira area, south–central Ethiopia: a multilevel analysis. *Malar J* 2013; **12**: 273.

70. Yamamoto S, Louis VR, Sie A, Sauerborn R. Household risk factors for clinical malaria in a semi–urban area of Burkina Faso: a case–control study. *Trans R Soc Trop Med Hyg* 2010; **104**: 61–5.

71. Ye Y, Hoshen M, Louis V, Seraphin S, Traore I, Sauerborn R. Housing conditions and *Plasmodium falciparum* infection: protective effect of iron–sheet roofed houses. *Malar J* 2006; **5**: 8.

72. Yukich JO, Taylor C, Eisele TP, et al. Travel history and malaria infection risk in a low–transmission setting in Ethiopia: a case control study. *Malar J* 2013; **12**: 33.

73. Zhou G, Munga S, Minakawa N, Githeko AK, Yan G. Spatial relationship between adult malaria vector abundance and environmental factors in western Kenya highlands. *Am J Trop Med Hyg* 2007; **77**: 29–35.

74. Ayele DG, Zewotir TT, Mwambi HG. Prevalence and risk factors of malaria in Ethiopia. *Malar J* 2012; **11**: 195.

75. Ayele DG, Zewotir TT, Mwambi HG. Spatial distribution of malaria problem in three regions of Ethiopia. *Malar J* 2013; **12**: 207.

76. Bell D, Bryan J, Cameron A, Fernando M, Leafasia J, Pholsyna K. Malaria in Honiara, Solomon Islands: reasons for presentation and human and environmental factors influencing prevalence. *Southeast Asian J Trop Med Public Health* 1997; **28**: 482–8.

77. Cano J, Descalzo MA, Moreno M, et al. Spatial variability in the density, distribution and vectorial capacity of anopheline species in a high transmission village (Equatorial Guinea). *Malar J* 2006; **5**: 21.

78. de Barros FS, Honorio NA, Arruda ME. Temporal and spatial distribution of malaria within an agricultural settlement of the Brazilian Amazon. *J Vector Ecol* 2011; **36**: 159–69.

79. Kibret S, Alemu Y, Boelee E, Tekie H, Alemu D, Petros B. The impact of a small–scale irrigation scheme on malaria transmission in Ziway area, Central Ethiopia. *Trop Med Int Health* 2010; **15**: 41–50.

80. Lwetoijera DW, Kiware SS, Mageni ZD, et al. A need for better housing to further reduce indoor malaria transmission in areas with high bed net coverage. *Parasites Vectors* 2013; **6**: 57.

81. Mala AO, Irungu LW, Shililu JI, et al. Plasmodium falciparum transmission and aridity: a Kenyan experience from the dry lands of Baringo and its implications for Anopheles arabiensis control. *Malar J* 2011; **10**: 121.

82. Manah AM, Shah SA, Hassan R, Ibrahim MY. The influence of environmental risk factors and individual behaviors on malaria occurrence in lahad datu district of sabah, Malaysia: A case control study. *Am J Trop Med Hyg* 2012; **87**: 107–8.

83. Muturi EJ, Shililu JI, Jacob BG, et al. Diversity of riceland mosquitoes and factors affecting their occurrence and distribution in Mwea, Kenya. *J Am Mosq Contr Assoc* 2008; **24**: 349–58.

84. Nkuo–Akenji T, Ntonifor NN, Ndukum MB, et al. Environmental factors affecting malaria parasite prevalence in rural Bolifamba, South West Cameroon. *Afr J Health Sci* 2006; **13**: 40–6.

85. Palsson K, Jaenson TG, Dias F, Laugen AT, Bjorkman A. Endophilic Anopheles mosquitoes in Guinea Bissau, west Africa, in relation to human housing conditions. *J Med Entomol* 2004; **41**: 746–52.

86. Somi MF, Butler JR, Vahid F, Njau J, Kachur SP, Abdulla S. Is there evidence for dual causation between malaria and socioeconomic status? Findings from rural Tanzania. *Am J Trop Med Hyg* 2007; **77**: 1020–7.

87. Somi MF, Butler JR, Vahid F, Njau JD, Kachur SP, Abdulla S. Use of proxy measures in estimating socioeconomic inequalities in malaria prevalence. *Trop Med Int Health* 2008; **13**: 354–64.

88. Subramanian S, Manoharan A, Sahu S, et al. Living conditions and occurrence of malaria in a rural community. *Indian J Malariol* 1991; **28**: 29–37.

89. Tilaye T, Deressa W. Prevalence of urban malaria and assocated factors in Gondar Town, Northwest Ethiopia. *Ethiopian Med J* 2007; **45**: 151–8.

90. van der Hoek W, Konradsen F, Dijkstra DS, Amerasinghe PH, Amerasinghe FP. Risk factors for malaria: a microepidemiological study in a village in Sri Lanka. *Trans R Soc Trop Med Hyg* 1998; **92**: 265–9.
