# Supplementary material for: The evidence for improving housing to reduce malaria: a systematic review and meta-analysis
Source: Malar J. 2015 Jun 9;14:209. doi: 10.1186/s12936-015-0724-1 (PMC4460721; doi:10.1186/s12936-015-0724-1)
Supplement: Additional file 3: — Risk of bias assessment for studies included in the quantitative analysis. [file 12936_2015_724_MOESM3_ESM.pdf]

Tusting LS, Ippolito M, et al. The evidence for improving housing to reduce malaria: a systematic review and meta-analysis.

Additional File 3. Risk of bias assessment

a. Risk of bias assessment for studies included in the quantitative analysis (intervention studies, n=6)

Reference	Allocation sequence generation	Allocation concealment	Baseline outcome measurements	Baseline features	Incomplete outcome data	Length of follow up	Blinding (performance)	Blinding (detection)	Contamination	Selective outcome reporting	Recruitment bias
**Kampango 2013**	Low	High	Unclear	Unclear	Low	High	High	High	Low	Low	Low
	Intervention randomly allocated	Patients and investigators could forsee assignment	No baseline measurement of outcome	No information reported	Low missing data	Follow up period less than one year or transmission season	Performance bias possible due to knowledge of the allocated interventions	Primary outcomes not assessed blinded.	Unlikely that the control group received the intervention	All pre–specified outcomes are reported	No change in size or number of clusters after randomisation
**Kirby 2009**	Low	High	Low	Low	Low	Low	High	Low	Low	Low	Low
	Intervention randomly allocated	Patients and investigators could forsee assignment	Outcomes were measured pre intervention and adjusted for in analysis	Baseline characteristics of the study and control areas are reported and similar	Low missing data, balanced across groups	Follow up period at least one transmission season	Performance bias possible due to knowledge of the allocated interventions	Primary outcomes assessed blinded	Unlikely that the control group received the intervention	All pre–specified outcomes are reported	No change in size or number of clusters after randomisation
**Massebo 2013**	Low	High	Low	Low	Low	High	High	High	Low	Low	Low
	Intervention randomly allocated	Patients and investigators could forsee assignment	Outcomes were measured pre intervention and no important differences were present	Baseline characteristics of the study and control areas are reported and similar	No missing data	Follow up period less than one year or transmission season	Performance bias possible due to knowledge of the allocated interventions	Primary outcomes not assessed blinded.	Unlikely that the control group received the intervention	All pre–specified outcomes are reported	No change in size or number of clusters after randomisation
**Mng'ong'o 2011**	Low	High	Unclear	High	Unclear	Low	High	High	Low	Low	Low
	Houses selected in stepwise fashion starting from random point	Patients and investigators could forsee assignment	No baseline measurement of outcome	Differences between control and intervention areas	No information reported	Follow up period at least one transmission season	Performance bias possible due to knowledge of the allocated interventions	Primary outcomes not assessed blinded.	Unlikely that the control group received the intervention	All pre–specified outcomes are reported	No change in size or number of clusters after randomisation
**Njie 2009**	Low	High	Unclear	Unclear	Low	High	High	High	Low	Low	Low
	Intervention randomly allocated	Patients and investigators could forsee assignment	No baseline measurement of outcome	No information	Low missing data	Follow up period less than one year or transmission season	Performance bias possible due to knowledge of the allocated interventions	Primary outcomes not assessed blinded.	Unlikely that the control group received the intervention	All pre–specified outcomes are reported	No change in size or number of clusters after randomisation
**Ogoma 2010**	Unclear	High	Unclear	Unclear	Low	High	High	High	Low	Low	Low
	No information reported	Patients and investigators could forsee assignment	No baseline measurement of outcome	No information reported	Low missing data	Follow up period less than one year or transmission season	Performance bias possible due to knowledge of the allocated interventions	Primary outcomes not assessed blinded.	Unlikely that the control group received the intervention	All pre–specified outcomes are reported	No change in size or number of clusters after randomisation

Tusting LS, Ippolito M, et al. The evidence for improving housing to reduce malaria: a systematic review and meta-analysis.

b. Risk of bias assessment for studies included in the quantitative analysis (case–control studies, n=14)

Reference	Selection				Comparability	Exposure			Overall quality assessment score (max 9)
	**Is the case definition adequate?**	**Representativeness of the cases**	**Selection of controls**	**Definition of controls**	**Comparability of cases and controls on the basis of the design or analysis**	**Ascertainment of exposure**	**Same method of ascertainment for cases and controls**	**Non–response rate**	
Adiamah 1993	Yes, with independent validation *	Consecutive or obviously representative series of cases *	Community controls *	No clinical malaria *	Study controls for age *	Structured interview and direct observation where blind to case/control status *	Yes *	Not described	7
Al–Taiar 2009	Yes, with independent validation *	Consecutive or obviously representative series of cases *	Community controls *	No history of malaria in past 6 months *	Study controls for age, location, area of residence, khat trees, larval habitats, IRS, history of travel**	Interview not blinded to case/control status	Yes *	Non respondents described	7
Brooker 2004	Yes, with independent validation *	Consecutive or obviously representative series of cases *	Community controls *	No malaria infection *	Study controls for age *	Structured interview where blind to case/control status *	Yes *	Not described	7
Butraporn 1986	Yes, record linkage	Consecutive or obviously representative series of cases *	Community controls *	No malaria infection *	Study controls for age, gender *	Structured interview where blind to case/control status *	Yes *	Not described	6
Coleman 2010	Yes, record linkage	Consecutive or obviously representative series of cases *	Community controls *	Households with no confirmed case of clinical malaria during the study period *	Study controls for household wealth *	Structured interview where blind to case/control status *	Yes *	Not described	6
Danis–Lozano 2007	Yes, with independent validation *	Potential for biases (not all incident cases were recruited, without explanation)	Community controls *	No malaria infection *	Study controls for age, village, occupation **	Structured interview where blind to case/control status *	Yes *	Rate different and no designation	7
Ernst 2009	Yes, record linkage	Consecutive or obviously representative series of cases *	Community controls *	No malaria symptoms or history of malaria (however not slide confirmed negative)	Study controls for age, study site **	Interview not blinded to case/control status	Yes *	Same rate for both groups *	6
Guthman 2001	Yes, with independent validation *	Consecutive or obviously representative series of cases *	Community controls *	No clinical malaria or infection*	Study controls for age, gender, area of residence, age of house, IRS in past six months, distance to nearest canal, agricultural work, education level **	Structured interview where blind to case/control status *	Yes *	No description	8
Koram 1995	Yes, with independent validation *	Consecutive or obviously representative series of cases *	Community controls *	No clinical malaria *	Study controls for age *	Structured interview and direct observation *	Yes *	No description	7
Ong'Echa 2006	Yes, with independent validation *	Consecutive or obviously representative series of cases *	Hospital controls	No malaria infection or history of fever in past 14 days *	Study controls for age, axillary temperature ≥37.5°C, wasting, caretaker education, occupation of household head and mother, bednet use, mosquito coil use **	Structured interview; blinding not described	Yes *	Same rate for both groups *	7
Siri 2010	Yes, with independent validation *	Consecutive or obviously representative series of cases *	Community controls *	No high parasitaemia or malaria anaemia *	Study controls for age, mosquito coils, bednet ownership, sleeping in rural area, household head gender, wealth, land ownership, domestic animals in residence, crowding, urbanisation **	Direct observation and interview not blind to case/control status	No	No description	6

Tusting LS, Ippolito M, et al. The evidence for improving housing to reduce malaria: a systematic review and meta-analysis.

b. Risk of bias assessment for studies included in the quantitative analysis (case–control studies, n=14) (continued).

Reference	Selection				Comparability	Exposure			Overall quality assessment score (max 9)
	Is the case definition adequate?	Representativeness of the cases	Selection of controls	Definition of controls	Comparability of cases and controls on the basis of the design or analysis	Ascertainment of exposure	Same method of ascertainment for cases and controls	Non–response rate	
Van der Hoek 2003	Yes, record linkage	Consecutive or obviously representative series of cases *	Community controls *	No malaria infection	Study controls for age, gender, distance to stream, distance to cattle shed, use of bednets, pyrethrum coils and traditional fumigants, IRS **	Direct observation; not clear whether blinded to case/control status	Yes *	No description	5
Yanamoto 2010	Yes, record linkage	Consecutive or obviously representative series of cases *	Community controls *	No description of malaria infection status	Study design controls for age *	Direct observation *	Yes *	No description	5
Yukich 2013	Yes, record linkage	Consecutive or obviously representative series of cases *	Hospital controls	No malaria infection *	Study does not control for other factors	Structured interview where blind to case/control status *	Yes *	No description	4

ITN: Insecticide-treated net; LLIN: Long-lasting insecticidal net; IRS: Indoor residual spraying

Tusting LS, Ippolito M, et al. The evidence for improving housing to reduce malaria: a systematic review and meta-analysis.

c. Risk of bias assessment for studies included in the quantitative analysis (cross–sectional studies, n=31)

Reference	Selection		Comparability	Exposure	Overall quality assessment score (max 5)
	**Representativeness of the sample**	**Assessment of outcome**	**Comparability of groups on the basis of the design or analysis**	**Ascertainment of exposure**	
Abe 2009	Truly representative of the average individual or household in the community *	Independent blind assessment *	Study controls for age, numebr of family members, bednet use**	Structured interview*	5
Al–Makhlafi 2011	Somewhat representative of the average individual or household in the community *	Independent blind assessment *	Study does not control for other factors	Structured interview*	3
Barber 1935	Truly representative of the average individual or household in the community *	Independent blind assessment *	Study does not control for other factors	Direct observation *	3
Bosman 1992	No description of the derivation of the sample	Independent blind assessment *	Study does not control for other factors	Secure record *	2
Bradley 2013	Truly representative of the average individual or household in the community *	Independent blind assessment *	Study controls for age, year of survey, spray coverage, net use, socioeconomic status, living in an urban area, crowding, eaves and screening **	Secure record *	5
Briggs–Watson	No description of the derivation of the sample	Independent blind assessment *	Study does not control for other factors	Direct observation *	2
Burkot 1989	No description of the derivation of the sample	Independent blind assessment *	Study does not control for other factors	Direct observation *	2
Charlwood 2003	Truly representative of the average individual or household in the community *	Independent blind assessment *	Study does not control for other factors	Direct observation *	3
Dahesh 2009	No description of the derivation of the sample	Independent blind assessment *	Study does not control for other factors	Structured interview *	2
de Almeida 2010	No description of the derivation of the sample	Independent blind assessment *	Study does not control for other factors	Structured interview *	2
de Beaudrap 2010	Somewhat representative of the average individual or household in the community (children aged 0–5 years) *	Independent blind assessment *	Study controls for age, weight–for–age, socioeconomic status, education level of household head, latitude, altitude, bednet use **	Structured interview *	5
Ekpenyong 2008	Somewhat representative of the average individual or household in the community (school children) *	Independent blind assessment *	Study does not control for other factors	Structured interview *	3
Geissbuhler 2007	Truly representative of the average individual or household in the community *	Independent blind assessment *	Study does not control for other factors	Direct observation *	3
Hagmann 2003	Truly representative of the average individual or household in the community *	Independent blind assessment *	Study does not control for other factors	Structured interview *	3
Hiscox 2013	Truly representative of the average individual or household in the community *	Independent blind assessment *	Study controls for village, location of kitchen, wall material, veranda style, presence of animals *	Visual observation *	4
Kaur 2009	Somewhat representative of the average individual or household in the community (77.5% response rate) *	Independent blind assessment *	Study controls for age, protective clothing, going out at night, ever staying in another village **	Structured interview *	5
Kirby 2008	Truly representative of the average individual or household in the community *	Independent blind assessment *	Study controls for distance to nearest pit latrine, horses in compound, eave type, crowding, churai in room *	Visual observation *	4
Magalhaes 2012	Truly representative of the average individual or household in the community *	Independent blind assessment *	Study does not control for other factors	Structured interview *	3
Malik 2003	Truly representative of the average individual or household in the community *	Independent blind assessment *	Study controls for age, gender, season, bednet use, distance to health facility, indoor breeding, region, IRS **	Structured interview *	5
Mmbando 2011	Truly representative of the average individual or household in the community *	Independent blind assessment *	Study controls for age, bednet use, socioeconomic status, passive case detection, altitude, season **	Structured interview *	5

Tusting LS, Ippolito M, et al. The evidence for improving housing to reduce malaria: a systematic review and meta-analysis.

c. Risk of bias assessment for studies included in the quantitative analysis (cross–sectional studies, n=31) (continued).

Reference	Selection		Comparability	Exposure	Overall quality assessment score (max 5)
	**Representativeness of the sample**	**Assessment of outcome**	**Comparability of groups on the basis of the design or analysis**	**Ascertainment of exposure**	
Osterbauer 2012	Selected group (infants recruited at antenatal clinic)	Independent blind assessment *	Study controls for age, HIV–exposure at birth, period of enrollment, gender, mother's age, bednet use, trimethoprim–sulphamethoxazole prophylaxis **	Structured interview *	4
Ouma 2007	Selected group (women attending antenatal clinic)	Independent blind assessment *	Study controls for age, ethnicity, area of residence, spending night in malarious area, trimester, bednet use, treatment of bednet **	Structured interview *	4
Pardo 2006	Truly representative of the average individual or household in the community *	Independent blind assessment *	Study does not control for other factors	Structured interview *	3
Rulisa 2013	Selected group (households in which one member presented to health facility with fever)	Independent blind assessment *	Study controls for gender, age, positivity of study index case, bednet ownership, main roof material, presence of open water vessel, vegetation around home, electricity **	Structured interview *	4
Sintasath 2005	Truly representative of the average individual or household in the community *	Independent blind assessment *	Study controls for ecological strata, eaves, IRS, distance to river, rainfall *	Structured interview *	4
Temu 2012	Truly representative of the average individual or household in the community *	Independent blind assessment *	Study controls for age, socioeconomic status, year of survey **	Structured interview *	5
Townes 2013	Truly representative of the average individual or household in the community *	Independent blind assessment *	Study does not control for other factors	Structured interview *	3
Winskill 2012	Truly representative of the average individual or household in the community *	Independent blind assessment *	Study does not control for other factors	Structured interview *	3
Wolff 2001	Selected group (households who received Habitat for Humanity homes and their neighbours)	Independent blind assessment *	Study controls for water source, occupation, education, malaria knowledge, waste disposal method *	Structured interview *	3
Woyessa 2013	Truly representative of the average individual or household in the community *	Independent blind assessment *	Study does not control for other factors	Structured interview *	3
Ye 2006	Truly representative of the average individual or household in the community *	Independent blind assessment *	Study controls for site, presence of larval habitat, well and animal enclosure, bednet use *	Structured interview *	4

ITN: Insecticide-treated net; LLIN: Long-lasting insecticidal net; IRS: Indoor residual spraying

Tusting LS, Ippolito M, et al. The evidence for improving housing to reduce malaria: a systematic review and meta-analysis.

d. Risk of bias assessment for studies included in the quantitative analysis (cohort studies, n=21)

Reference	Selection			Comparability	Exposure			Overall quality assessment score (max 9)
	**Representativeness of the sample**	**Selection of the non–exposed cohort**	**Assessment of outcome**	**Comparability of groups on the basis of the design or analysis**	**Ascertainment of exposure**	**Was follow–up at least one transmission season or year?**	**Adequacy of follow up of cohorts**	
Animut 2013	Truly representative of the average individual or household in the community *	Drawn from the same community as the exposed cohort *	Independent blind assessment *	Study does not control for other factors	Direct observation *	Yes *	Complete follow up *	6
Asante 2013	Somewhat representative of the average individual or household in the community (infants only) *	Drawn from the same community as the exposed cohort *	Independent blind assessment *	Study controls for age, mother's gravidity, primagravidae, wealth, maternal anaemia, urban or rural, distance to health facility, ITN use, malaria exposure score (based on sibling and neighbour malaria antibody) **	Direct observation *	Yes *	Subjects lost to follow up unlikely to introduce bias – small number lost (83.2%) *	8
Coogle 1927	No description of the derivation of the cohort	No description of the derivation of the unexposed cohort	No description	Study does not control for other factors	No description	No description	No statement	0
Gamage–Mendis 1991	Truly representative of the average individual or household in the community *	Drawn from the same community as the exposed cohort *	Independent blind assessment *	Study does not control for other factors	Direct observation *	Yes *	No statement	5
Ghebreyseus 2000	Somewhat representative of the average individual or household in the community (children aged 0–10 years) *	Drawn from the same community as the exposed cohort *	Independent blind assessment *	Study adjusts for age, sex, time at risk, eave type, presence of windows, number of sleeping rooms, livestock ownership, radio ownership, water source, use of irrigated land **	Structured interview *	Yes *	Complete follow up *	9
Haque 2013	Truly representative of the average individual or household in the community *	Drawn from the same community as the exposed cohort *	Record linkage *	Study controls for bednet ratio, house density, distance to nearest streams, elevation *	Visual observation *	Yes *	No statement	6
Hustache 2007	Somewhat representative of the average individual or household in the community *	Drawn from the same community as the exposed cohort *	Record linkage *	Study does not control for other factors	Direct observation *	Yes *	No statement	5
Konradsen 2000	Truly representative of the average individual or household in the community *	Drawn from the same community as the exposed cohort *	Independent blind assessment *	Study does not control for other factors	Direct observation *	Yes *	Complete follow up *	6
Kreuels 2008	Somewhat representative of the average individual or household in the community (infants enrolled into IPTi trial) *	Drawn from the same community as the exposed cohort *	Independent blind assessment *	Study controls for village of residence *	Structured interview *	Yes *	No statement	6
Lindsay 1988	No description of the derivation of the cohort	Drawn from the same community as the exposed cohort *	No description	Study does not control for other factors	No description	Yes *	No statement	2

Tusting LS, Ippolito M, et al. The evidence for improving housing to reduce malaria: a systematic review and meta-analysis.

d. Risk of bias assessment for studies included in the quantitative analysis (cohort studies, n=21) (continued).

Reference	Selection			Comparability	Exposure			Overall quality assessment score (max 5)
	**Representativeness of the sample**	**Selection of the non–exposed cohort**	**Assessment of outcome**	**Comparability of groups on the basis of the design or analysis**	**Ascertainment of exposure**	**Was follow–up at least one transmission season or year?**	**Adequacy of follow up of cohorts**	
Lindsay 1988	Truly representative of the average individual or household in the community *	Drawn from the same community as the exposed cohort *	Independent blind assessment *	Study does not control for other factors	Direct observation *	Yes *	Complete follow up *	6
Liu 2014	Somewhat representative of the average individual in the community (infants enrolled into IPTi trial) *	Drawn from the same community as the exposed cohort *	Independent blind assessment *	Study controls for age, mother's education, wealth index, bednet and repellent use, water source, electricity, urban or rural, IPTi trial arm **	Direct observation *	Yes *	Complete follow up *	8
Maheu–Giroux 2009	Somewhat representative of the average individual in the community (90% residents consented to participate) *	Drawn from the same community as the exposed cohort *	Record linkage *	Study does not control for other factors	Structured interview *	Yes *	Follow up rate <80% and no description of those lost	5
Mututi 2008	Truly representative of the average individual or household in the community *	Drawn from the same community as the exposed cohort *	Independent blind assessment *	Study does not control for other factors	Direct observation *	Yes *	Complete follow up *	6
Nahum 2010	Truly representative of the average individual or household in the community *	Drawn from the same community as the exposed cohort *	Independent blind assessment *	Study controls for sex, site, bed type, bednet use, pirogue, fishing net *	Structured interview *	Yes *	Subjects lost to follow up unlikely to introduce bias (small number lost) *	7
Oesterholt 2006	Truly representative of the average individual or household in the community *	Drawn from the same community as the exposed cohort *	Record linkage *	Study adjusts for age *	Structured interview *	Yes *	Complete follow up *	7
Peterson 2009a	Truly representative of the average individual or household in the community *	Drawn from the same community as the exposed cohort *	Record linkage *	Study does not control for other factors	Structured interview *	No	No statement	4
Peterson 2009b	Truly representative of the average individual or household in the community *	Drawn from the same community as the exposed cohort *	Record linkage *	Study controls for bednet ownership, vegetation in compound, distance to larval habitats, temperature, rainfall, larval densities*	Direct observation *	No	No statement	5
Russell 2013	Truly representative of the average individual or household in the community *	Drawn from the same community as the exposed cohort *	Independent blind assessment *	Study does not control for other factors	Direct observation *	Yes *	Complete follow up *	6
Wanzirah 2015	Truly representative of the average individual or household in the community *	Drawn from the same community as the exposed cohort *	Independent blind assessment *	Study controls for age, gender, household wealth **	Direct observation *	Yes *	Subjects lost to follow up unlikely to introduce bias (>80% follow–up)*	8
Zhou 2007	Truly representative of the average individual or household in the community *	Drawn from the same community as the exposed cohort *	Independent blind assessment *	Study does not control for other factors	Direct observation *	Yes *	Complete follow up *	6

ITN: Insecticide-treated net; LLIN: Long-lasting insecticidal net; IRS: Indoor residual spraying; IPTi: intermittent preventive treatment in infants.
