# Supplementary material for: The evidence for improving housing to reduce malaria: a systematic review and meta-analysis
Source: Malar J. 2015 Jun 9;14:209. doi: 10.1186/s12936-015-0724-1 (PMC4460721; doi:10.1186/s12936-015-0724-1)
Supplement: Additional file 4: — Funnel plots to assess publication bias. [file 12936_2015_724_MOESM4_ESM.pdf]

Additional File 4. Funnel plots to assess publication bias in the meta–analysis of modern versus traditional housing.

A

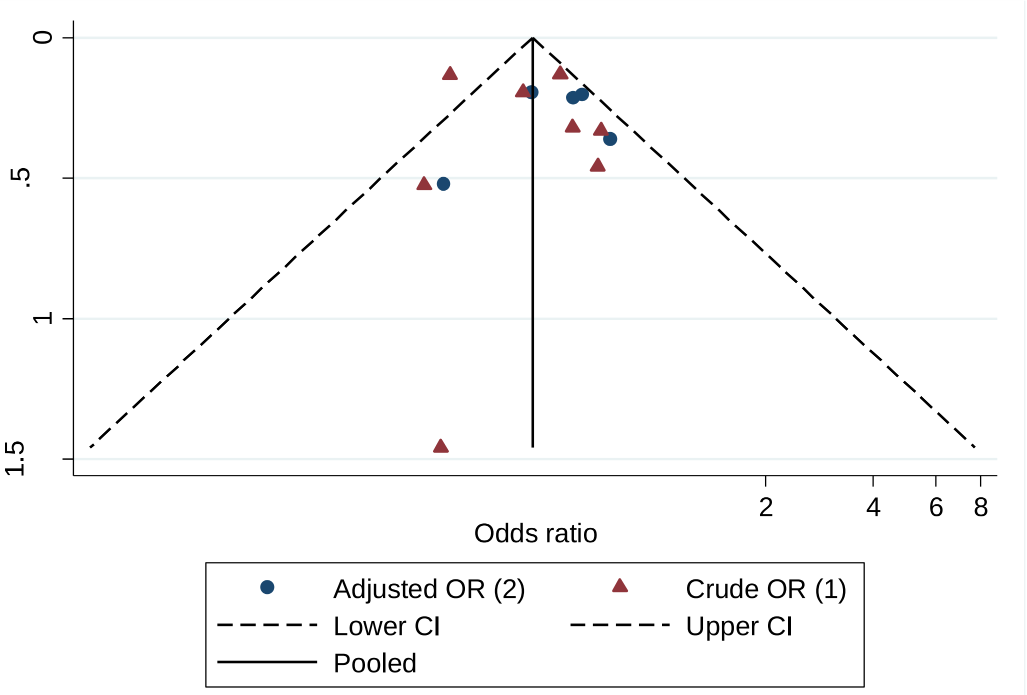

B

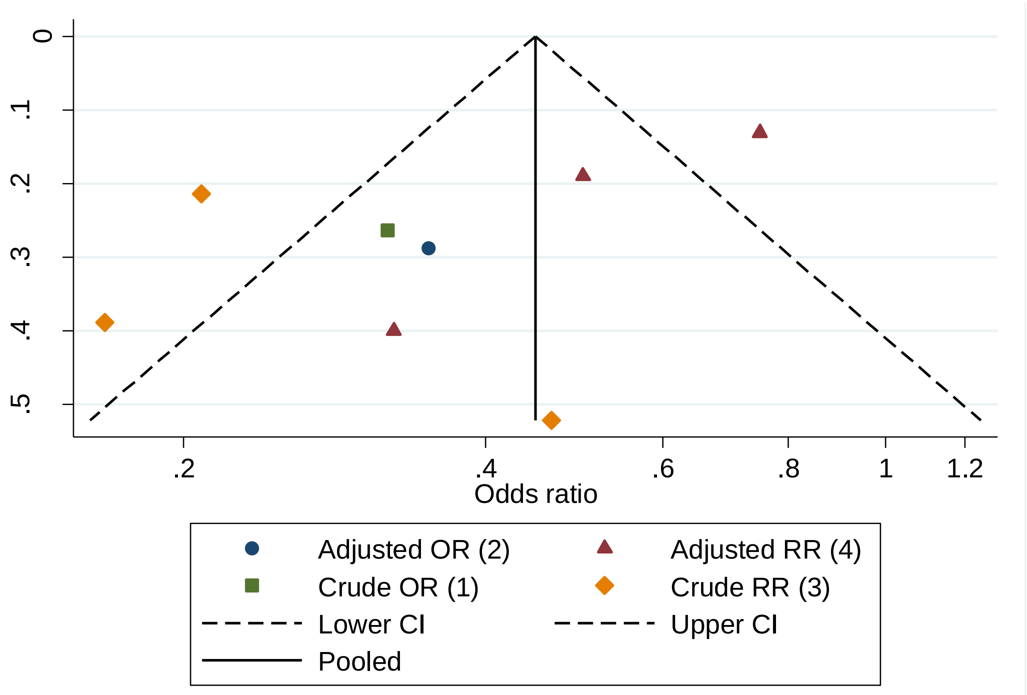

Funnel plots to assess publication bias in the meta–analysis of modern versus traditional housing.
Plots show study size as a function of effect size for studies included. Asymmetry is indicative of publication bias, selective outcome reporting, small-study effects, or selective analysis reporting. (A) Studies reporting malaria infection. 1: Case–control, cross–sectional and cohort studies: crude odds ratio, 2: Case–control, cross–sectional and cohort studies: adjusted odds ratio. (B) Studies reporting clinical malaria. 1: Case–control and cross–sectional studies (crude odds ratio), 2: Case–control and cross–sectional studies (adjusted odds ratio), 3: Cohort studies (crude rate ratio), 4: Cohort studies (adjusted rate ratio).
