# Supplementary material for: The evidence for improving housing to reduce malaria: a systematic review and meta-analysis
Source: Malar J. 2015 Jun 9;14:209. doi: 10.1186/s12936-015-0724-1 (PMC4460721; doi:10.1186/s12936-015-0724-1)
Supplement: Additional file 5: — Association between house construction and entomological outcomes. [file 12936_2015_724_MOESM5_ESM.pdf]

Tusting LS, Ippolito M, et al. The evidence for improving housing to reduce malaria: a systematic review and meta-analysis.

Additional File 5. Association between house construction and entomological outcomes

a. Intervention studies

Study reference	Design	Intervention (type of screening)	Comparison	Outcome	Measurement of outcome	Mean density or rate				Measure of effect	
						Pre–intervention		Post–intervention			
						Control	Treatment	Control	Treatment		
**Massebo 2013**	RCS (pilot)	Full	Screening vs no screening	HBR	Mean number *An. arabiensis* per CDC light trap per night	20.1 (10.9–29.3)	20.3 (12.8– 27.8)	7.9 (6.5–10.1)	4.8 (3.9–6.2)	Abundance ratio	0.61 (0.44-0.83)
**Mng'ong'o 2011**	RCS (pilot)	Full	Lantana plant vs no Lantana plant	HBR	Mean number adult anophelines per CDC light trap per night	n/a	n/a	–	–	Crude IRR	0.54 (0.40–0.73)
**Mng'ong'o 2011**	RCS (pilot)	Full	Lantana plant vs no Lantana plant	HBR	Mean number adult anophelines per CDC light trap per night	n/a	n/a	–	–	IRR adjusted for smoke stains	0.503 (0.380–0.667)
**Ogoma 2010**	Non–randomized cross–over study (pilot study)	Eaves	Screened eaves vs no screening	HBR	Mean number *An. gambiae* s.l. per CDC light trap per night	n/a	n/a	80.0 (4–630)	59.0 (9–415)	Relative Rate	0.91 (0.84–0.98)
**Ogoma 2010**	Non–randomized cross–over study (pilot)	Windows	Screened windows vs no screening	HBR	Mean number *An. gambiae* s.l. per CDC light trap per night	n/a	n/a	80.0 (4–630)	80.0 (15–370)	Relative Rate	0.98 (0.94–1.02)
**Ogoma 2010**	Non–randomized cross–over study (pilot)	Door	Screened doors vs no screening	HBR	Mean number *An. gambiae* s.l. per CDC light trap per night	n/a	n/a	80.0 (4–630)	96.0 (17–700)	Relative Rate	1.03 (0.97–1.09)
**Kirby 2009**	RCS	Ceiling	Ceiling vs no screening	HBR	Mean number *An. gambiae* s.l. per CDC light trap per night	n/a	n/a	37.5 (31.6–43.3)	19.1 (16.1–22.1)	Ratio of means for total *An. gambiae* s.l. over all trapping visits, adjusted for location, year, SES, wall material, horses, people in house	0.60 (0.46–0.80)
**Kirby 2009**	RCS	Full	Full vs no screening	HBR	Mean number *An. gambiae* s.l. per CDC light trap per night	n/a	n/a	37.5 (31.6–43.3)	15.2 (12.9–17.4)	Ratio of means for total *An. gambiae* s.l. over all trapping visits, adjusted for location, year, SES, wall material, horses, people in house	0.46 (0.34–0.63)
**Kirby 2009 (2006 data)**	RCS	Ceiling	Ceiling vs no screening	EIR	Measured using CDC light traps	n/a	n/a	2.27 (1.38–3.16)	1.14 (0.85–1.42)	Abundance ratio	0.50 (0.32-0.79)
**Kirby 2009 (2006 data)**	RCS	Full	Full vs no screening	EIR	Measured using CDC light traps	n/a	n/a	2.27 (1.38–3.16)	0.77 (0.57–0.96)	Abundance ratio	0.34 (0.21-0.54)
**Kirby 2009 (2007 data)**	RCS	Ceiling	Ceiling vs no screening	EIR	Measured using CDC light traps	n/a	n/a	1.35 (0.74–1.97)	0.90 (0.22–1.57)	Abundance ratio	0.67 (0.28-1.58)
**Kirby 2009 (2007 data)**	RCS	Full	Full vs no screening	EIR	Measured using CDC light traps	n/a	n/a	1.35 (0.74–1.97)	0.42 (0.24–0.63)	Abundance ratio	0.31 (0.16-0.59)
**Kampango 2013**	RCS (pilot)	Eaves	Gables screened with local cloth vs no screening	HBR	Mean number *An. funestus* per CDC light trap per night	n/a	n/a	43.4 (38.0–49.6)	13.0 (10.7–15.7)	Crude IRR	0.3 (0.25–0.37)
**Kampango 2013**	RCS (pilot)	Full	Gables screened with local cloth vs no screening	HBR	Mean number *An. gambiae* s.l. per CDC light trap per night	n/a	n/a	6.88 (4.98–9.51)	2.13 (1.48–3.08)	Crude IRR	0.31 (0.19–0.50)
**Njie 2009**	Randomised cross–over study (pilot)	Eaves	Screened eaves vs no screening	HBR	Mean number *An. gambiae* s.l. per CDC light trap per night	n/a	n/a	6.1 (3.5–10.0)	2.1 (1.3–3.1)	Percent reduction	0.34 (0.18-0.67)
**Njie 2009**	Randomised cross–over study (pilot)	Screened eaves	Screened eaves vs no screening	Adult density	Odds of finding *An.gambiae* in house (CDC light trap)	n/a	n/a	–	–	OR adjusted for trapping week, crossover group, numbers of horses and cows in compounds	0.34 (0.20–0.56)

Tusting LS, Ippolito M, et al. The evidence for improving housing to reduce malaria: a systematic review and meta-analysis.

b. Observational studies

Reference	House feature	Specific comparison	Outcome	Mean density or rate		Measure of effect	Crude results	Adjusted results	Factors adjusted for
				Exposed	Unexposed				
**Barber 1935**	House type	New (tiled roof, ceiling, non–leaky) vs old (thatched roof, reed or no ceiling, in poor condition)	Density of adult anophelines (mean number *An. elutus* and *An. maculipennis* per resting catch)	6.8	9.4	None reported	–	–	n/a
**Bosman 1992**	House type	Modern vs traditional	Density of adult Anophelines (August monthly mean number adult Anopheles per pyrethrum spray catch)	–	–	None reported	–	–	n/a
**Gamage–Mendis 1991**	House type	Poor vs good	Indoor resting density (geometric mean number of *Anopheles* per trap per night)	3.42	1.95	None reported	–	–	n/a
**Hiscox 2013**	Village type	Modern vs traditional homes	Human biting rate (mean number of *Anopheles* per CDC light trap per night)	–	–	IRR	0.72 (0.45–1.14)		n/a
**Konradsen 2000**	House type	Poor vs good	Presence (vs absence) of *An. culicifacies* in each collection (pyrethrum spray catch)	–	–	OR	1.6 (1.1–2.1)	–	n/a
**Konradsen 2000**	House type	Poor vs good	Presence (vs absence) of *An. subpictus* in each collection (pyrethrum spray catch)	–	–	OR	1.4 (1.1–1.7)	–	n/a
**Liu 2014**	House type	Highest quintile of housing index compared to lowest quintile (based on roof, wall and floor material, ceiling, eaves, screening)	Density of adult anophelines (mean number of *Anopheles* collected per household)	–	–	IRR	0.334 (0.228–0.489)	0.571 (0.373–0.874)	Cattle near house, water source, electricity, urban or rural
**Mutuku 2011**	House type	Poor vs good	Density of adult Anophelines (pyrethrum spray catch)	–	–	IRR	1.07 (0.72–1.44)	–	n/a
**Wanzirah 2015**	House type	Modern vs traditional	Human biting rate (mean number of *Anopheles* per CDC light trap per night)	–	–	IRR	–	0.48 (0.37–0.64)	Study site, household wealth
**Coogle 1927**	Screening	Screened vs unscreened	Density of adult anophelines (mean number *An. quadrimaculatus* per home	2.2	–	None reported	–	–	n/a
**Geissbuhler 2007**	Screening	No ceiling and unscreened windows vs ceiling and screned windows	Human biting rate (mean number of bites received by those sleeping indoors (HLC))	4.4	2.3	None reported	–	–	–
**Zhou 2007**	Screening	No screening vs screening	Density of adult anophelines (mean number of *An. gambiae* s.s. per house (pyrethrum spray catch))	4.15 (3.95–5.35)	2.92 (2.08–3.76)	OR (proportion houses with *An. gambiae* s.s.)	1.04 (1.01–1.07)	–	n/a
**Zhou 2007**	Screening	No screening vs screening	Density of adult anophelines (mean number of *An. funestus* per house (pyrethrum spray catch))	0.52 (0.37–0.67)	0.44 (0.29–0.59)	OR (proportion houses with *An. funestus*)	1.14 (1.09–1.20)	–	n/a

b. Observational studies (continued)

Reference	House feature	Specific comparison	Outcome	Mean density or rate		Measure of effect	Crude results	Adjusted results	Factors adjusted for
				Exposed	Unexposed				
**Adiamah 1993**	Main wall material	Mud vs brick/concrete	Density of adult Anophelines (geometric mean number adult *Anopheles* per light trap catch)	24.6	15.5	None reported	–	–	n/a
**Hiscox 2013**	Main wall material	Other vs wood	Human biting rate (mean number of *Anopheles* per CDC light trap per night)	–	–	IRR	1.83 (1.14–2.93)	2.35 (1.30–4.23)	Village, location of kitchen, veranda style, presence of animals
**Kirby 2008**	Main wall material	Mud vs cement	Human biting rate (mean number of *Anopheles* per CDC light trap per night)	–	–	IRR	5.36 (3.92–7.31)	1.44 (1.10–1.87)	Distance to nearest pit latrine, horses, eave type, crowding, churai in room
**Zhou 2007**	Main wall material	Mud vs brick	Density of adult anophelines (mean number of *An. gambiae* s.s. per house (pyrethrum spray catch))	4.16 (3.24–5.08)	0.86 (0.53–1.19)	OR (proportion houses with An. gambiae s.s.)	1.34 (1.29–1.40)	–	n/a
**Zhou 2007**	Main wall material	Mud vs brick	Density of adult anophelines (mean number of *An. funestus* per house (pyrethrum spray catch))	0.53 (0.37–0.65)	0.22 (0.06–0.38)	OR (proportion houses with *An. funestus*)	1.87 (1.70–2.04)	–	n/a
**Wanzirah 2015**	Main wall material	Cement, wood or metal vs mud	Human biting rate (mean number of *Anopheles* per CDC light trap per night)	–	–	IRR	–	0.63 (0.48–0.84)	Study site, household wealth
**Hiscox 2013**	Main roof material	Other vs iron	Human biting rate (mean number of *Anopheles* per CDC light trap per night)	–	–	IRR	0.49 (0.16–1.44)	–	n/a
**Kirby 2008**	Main roof type	Thatch vs metal	Human biting rate (mean number of *Anopheles* per CDC light trap per night)	–	–	IRR	1.15 (0.94–1.41)	–	n/a
**Wanzirah 2015**	Main roof material	Tiles or metal vs thatch	Human biting rate (mean number of *Anopheles* per CDC light trap per night)	–	–	IRR	–	0.72 (0.52–1.00)	Study site, household wealth
**Zhou 2007**	Main roof material	Grass thatch vs iron sheet	Density of adult anophelines (mean number of *An. gambiae* s.s. per house (pyrethrum spray catch))	4.26 (3.15–5.37)	2.00 (1.33–2.67)	OR (proportion houses with *An. gambiae* s.s.)	1.15 (1.12–1.19)	–	n/a
**Zhou 2007**	Main roof material	Grass thatch vs iron sheet	Density of adult anophelines (mean number of *An. funestus* per house (pyrethrum spray catch))	0.39 (0.27–0.51)	0.61 (0.39–0.83)	OR (proportion houses with *An. funestus*)	0.75 (0.71–0.79)	–	n/a
**Wanzirah 2015**	Main floor material	Woods, bricks or cement vs earth, sand, dung or stones	Human biting rate (mean number of *Anopheles* per CDC light trap per night)	–	–	IRR	–	0.42 (0.32–0.55)	Study site, household wealth
**Adiamah 1993**	Eaves	Presence vs absence of eaves	Density of adult Anophelines (geometric mean number adult *Anopheles* per light trap catch)	29.3	14.6	None reported	–	–	n/a
**Animut 2013**	Eaves	Open vs closed eaves	Density of *An. arabiensis* (mean number *An. arabiensis* per CDC light trap per house)	0.97 (0.60–1.34)	0.66 (0.43–0.88)	Abundance Ratio	1.5 (0.9-2.4)	–	n/a

b. Observational studies (continued)

Reference	House feature	Specific comparison	Outcome	Mean density or rate		Measure of effect	Crude results	Adjusted results	Factors adjusted for
				Exposed	Unexposed				
**Animut 2013**	Eaves	Open vs closed eaves	Density of *An. arabiensis* (mean number *An. arabiensis* per pyrethrum spray catch)	5.67 (4.22–7.12)	0.77 (–0.15–1.69)	None reported	7.4 (2.2-24.4)	–	n/a
**Kirby 2008**	Eaves	Closed vs open	Human biting rate (mean number of *Anopheles* per CDC light trap per night)	–	–	IRR	0.38 (0.32–0.46)	–	n/a
**Kirby 2008**	Eaves	Continuous variable (eave gap size)	Human biting rate (mean number of *Anopheles* per CDC light trap per night)	–	–	IRR	1.06 (1.04–1.08)	0.71 (0.60–0.85)	Distance to nearest pit latrine, horses, main wall material, crowding, churai in room
**Lindsay 1988**	Eaves	Open vs closed	Density of adult Anophelines (mean number of *Anopheles* per night)	–	–	Percent reduction	43.2	–	n/a
**Lindsay 1995 (wet season)**	Eaves	Open vs closed	Density of of adult anophelines (mean number *An. gambiae* s.l. caught under bednets)	–	–	Percent increase	–	10 (0–21)	Store room, bednets tucked, fire, ceiling
**Russell 2013**	Eaves	Closed vs open	Density of aduly *An. gambiae* s.l. (CDC light trap))	31.25% households in low anopheline density cluster (Z scores <1.96) had closed eaves vs 0% houses in high anopheline density cluster (Z scores >1.96)					
**Wanzirah 2015**	Eaves	Closed vs open	Human biting rate (mean number of *Anopheles* per CDC light trap per night)	–	–	IRR	–	0.58 (0.45–0.74)	Study site, household wealth
**Adiamah 1993**	Ceiling	No ceiling vs ceiling	Density of adult Anophelines (geometric mean number adult *Anopheles* per light trap catch)	20.5	10.7	None reported		–	n/a
**Lindsay 1995 (wet season)**	Ceiling	Ceiling present vs absent	Density of of adult anophelines (mean number *An. gambiae* s.l. caught under bednets)	–	–	Percent decrease	–	13 (1–25)	Store room, bednets tucked, fire, open eaves
**Burkot 1989**	Elevation	Houses built on stilts vs at ground level	Human biting rate (mean number of Anopheles per human landing catch)	50.8 (SD 46.9)	166 (SD 125)	None reported	–	–	–
**Charlwood 2003**	Elevation	Ground level homes vs homes built on stilts	Human biting rate (mean number of Anopheles per man hour of collection (HLC))	3.58 (2.9–4.4)	2.38 (1.7–3.3)	Abundance Ratio	1.5 (1.0-2.2)	–	n/a
**Charlwood 2003**	Elevation	Ground level homes vs homes built on stilts	Human biting rate (mean number of *An. gambiae* per light trap per night)			None reported	–	–	n/a
**Hiscox 2013**	Elevation	Continuous variable (height on stilts)	Human biting rate (mean number of *Anopheles* per CDC light trap per night)	–	–	IRR	1.00 (0.99–1.00)	–	n/a
**Animut 2013**	Windows	Present vs absent	Density of *An. arabiensis* (mean number *An. arabiensis* per CDC light trap per house)	0.27 (–0.04–0.60)	1.02 (0.78–1.27)	Abundance Ratio	0.3 (0.1-0.9)	–	n/a

b. Observational studies (continued)

Reference	House feature	Specific comparison	Outcome	Mean density or rate		Measure of effect	Crude results	Adjusted results	Factors adjusted for
				Exposed	Unexposed				
**Animut 2013**	Windows	Present vs absent	Density of *An. arabiensis* (mean number *An. arabiensis* per pyrethrum spray catch)	1.95 (0.72–3.18)	2.35 (1.30–3.39)	Abundance Ratio	0.8 (0.4-1.8)	–	n/a
**Hiscox 2013**	Doors and windows	Other covering vs resettlement style shutters	Human biting rate (mean number of *Anopheles* per CDC light trap per night)	–	–	IRR	1.23 (0.75–2.01)		n/a
**Hiscox 2013**	Doors and windows	Open vs resettlement style covers	Human biting rate (mean number of *Anopheles* per CDC light trap per night)	–	–	IRR	0.87 (0.25–3.12)		n/a
**Animut 2013**	Roof condition	Hole present vs absent	Density of *An. arabiensis* (mean number *An. arabiensis* per pyrethrum spray catch)	4.81 (3.31–6.31)	1.17 (0.24–2.10)	Abundance Ratio	4.1 (1.8-9.5)	–	n/a
**Animut 2013**	Roof condition	Hole present vs absent	Density of *An. arabiensis* (mean number *An. arabiensis* per CDC light trap per house)	1.12 (0.75–1.50)	0.61 (0.38–0.83)	Abundance Ratio	1.8 (1.1-3.0)	–	n/a
**Animut 2013**	Wall condition	Hole present vs absent	Density of *An. arabiensis* (mean number *An. arabiensis* per CDC light trap per house)	0.77 (0.54–1.01)	0.67 (0.32–1.02)	Abundance Ratio	1.1 (0.6-2.1)	–	n/a
**Animut 2013**	Wall condition	Hole present vs absent	Density of *An. arabiensis* (mean number *An. arabiensis* per pyrethrum spray catch)	3.27 (2.22–4.32)	0.73 (–0.48–1.94)	Abundance Ratio	4.5 (0.9-23.4)	–	n/a
**Hiscox 2013**	Veranda style	Closed vs open	Human biting rate (mean number of *Anopheles* per CDC light trap per night)	–	–	IRR	0.32 (0.18–0.58)	0.51 (0.23–1.11)	Village, location of kitchen, wall material, presence of animals

CDC: Centers for Disease Control and Prevention; OR: Odds Ratio; IRR: Incidence Rate Ratio; SES: socioeconomic status
